# Decorated bodies for eternal life: A multidisciplinary study of late Roman Period stucco-shrouded portrait mummies from Saqqara (Egypt)

**DOI:** 10.1371/journal.pone.0240900

**Published:** 2020-11-04

**Authors:** Stephanie Zesch, Manuela Gander, Marc Loth, Stephanie Panzer, M. Linda Sutherland, Adel H. Allam, Ibrahem Badr, Gregory S. Thomas, Saskia Wetzig, Albert Zink, Wilfried Rosendahl

**Affiliations:** 1 German Mummy Project, Reiss-Engelhorn-Museen, Mannheim, Germany; 2 Sculpture Collection Until 1800, Dresden State Art Collections, Dresden, Germany; 3 Department of Radiology, Trauma Center Murnau, Murnau, Germany; 4 Institute of Biomechanics, Trauma Center Murnau and Paracelsus Medical University Salzburg, Murnau, Germany; 5 MemorialCare Health System, Fountain Valley, California, United States of America; 6 Al-Azhar University, Cairo, Egypt; 7 Antiquities Restoration & Conservation Department, Faculty of Archaeology and Tourism Guidance, Misr University for Science and Technology, 6^th^ of October City, Egypt; 8 MemorialCare Heart & Vascular Institute, Southern California, and University of California, Irvine, California, United States of America; 9 Institute for Mummy Studies, Eurac Research, Bolzano, Italy; 10 Curt-Engelhorn-Centre Archaeometry gGmbH, Mannheim, Germany; University of Florence, ITALY

## Abstract

This study focuses on the multidisciplinary investigation of three stucco-shrouded mummies with mummy portrait from Egypt dating from the late 3^rd^ to the middle of the 4^th^ century AD, corresponding to the late Roman Period. These three mummies were excavated in the early 17^th^ and late 19^th^ centuries in the Saqqara necropolis near the ancient Egyptian capital of Memphis. Two of them experienced an interesting collection history, when they became part of the collection of the Elector of Saxony and King of Poland August II in Dresden, Germany, in 1728. The investigation includes information about the mummies’ discovery, collection history and shroud decoration obtained through Egyptological expertise. In addition, information on the state of preservation, technique of artificial mummification, age at death, sex, body height and health of the deceased was achieved through computed tomography (CT) analysis. Research yielded an adult male, a middle-aged female and a young female. Due to the rather poorly preserved bodies of the male and middle-aged female, a specific technique of artificial mummification could not be ascertained. Brain and several internal organs of the well-preserved young female were identified. Wooden boards, beads of necklaces, a hairpin, and metal dense items, such as lead seals, nails and two coins or medallions were discovered. Paleopathological findings included carious lesions, Schmorl’s nodes, evidence of arthritis and a vertebral hemangioma. The study revealed insights on the decoration and burial preparation of individuals of upper socioeconomic status living in the late Roman Period, as well as comprehensive bioanthropological information of the deceased.

## Introduction

An initial broad interest in Egyptian mummies arose in Europe during the 12^th^ century. As discussed by Dannenfeldt [1], this interest was based on misleading translations of early Arab medical texts by Western authors during the Medieval Period. These misleading translations led scholars to assume that as a substitute for natural bituminous materials for therapeutic purposes, as described by the early Arab physicians, embalmed bodies of ancient Egyptians could be used for medical therapy in the absence of true bitumen. As a result, Egyptian mummies were crushed and used as medicinal remedies, so-called *Mumia Aegyptiaca*, in European medicine. Prescribing the intake of pulverized Egyptian mummies with alleged healing power was fully established during the 16^th^ century, although, at the same time, early doubts had been raised by some scholars concerning the supposed medical efficiency of *Mumia Aegyptiaca* [1].

In the 16^th^ century, visitors to Egypt from the western cultural origin developed a particular interest for mummies as curiosities. In the 19^th^ century, the fascination of both the general public and scientists for ancient Egyptian culture increased significantly, mainly as a consequence of the Egyptian expedition of Napoleon Bonaparte (1798−1801). European travelers and scholars appropriated ancient artefacts to a great extent during their journeys through Egypt, including mummies and mummy parts of humans and animals. As a result, large numbers of mummies were taken out of Egypt. Many mummies were unwrapped out of curiosity to see what was beneath the textile wrappings, either in the course of particular social events for high-society people, so-called *Mummy parties*, but also by scientists in the absence of non-invasive methods of investigation [2].

Human skeletal remains and mummies are currently appreciated as valuable bioarcheological archives. Their scientific investigation enables exploration of the death cult including funerary tradition, body treatment and afterlife concepts, as well as the ability to generate biological profiles of ancient populations on questions such as provenance, mobility, migration, familial kinship relations, dietary habits, age at death, sex, body height, medical care and diseases. Those exceptional insights into lifestyles, living conditions and religious concepts of people from the past have been broadly demonstrated through multidisciplinary investigations of mummies originating from different cultures, time periods, geographic regions and climatic environments [3–14].

With regard to mummification tradition in Egypt during the Roman Period (30 BC−395 AD) [15], different types of elaborately decorated mummies are known which have been described in detail in other studies [2, 16–19]. These Roman Period mummies include portrait mummies which incorporate a portrait of the deceased on the exterior of the outermost wrapping. Depending upon the type of outermost wrapping, portrait mummies can be characterized into at least three distinct groups: rhomboid-wrapped, red-shrouded and stucco-shrouded [20]. Past studies on portrait mummies are limited concerning the number of specimens analyzed [20–24]. Radiological and physical anthropological investigations broadly consider contemporaneous customs of burial and body treatment to interpret findings in a multidisciplinary manner [25–29].

The current study focuses on three late Roman Period stucco-shrouded portrait mummies from the Saqqara necropolis near the ancient Egyptian capital of Memphis, located c. 25 kilometers south of the modern city of Cairo. As far as the authors know, these three stucco-shrouded portrait mummies are the only specimens with this type of mummy decoration that are still preserved with shroud and mummy intact from the Saqqara necropolis. Several previous publications have discussed their beautiful impressive shrouds, their archaeological discovery and interesting collection history [30–34]. Two of them, discovered in 1615, are considered the earliest examples of portrait mummies to have become known in Europe and remained the only known representatives of portrait mummies until the early 19^th^ century [32, 34].

However, a multidisciplinary investigation comprising the information on the collection history and iconographic details of the shrouds yielded by Egyptological analyses, and results on the technique of artificial mummification, state of bone and soft tissue preservation, the presence of foreign objects, age at death, sex, body height, anatomical anomalies and paleopathological findings achieved by evaluating CT data, is presented for the first-time in this study. This investigation considers cultural specifics; including aspects of death cult, burial customs and the preparation of bodies, as well as bioanthropological information; such as demographic profiles and health status of the deceased, thereby expanding the knowledge on burial customs and living conditions at the end of the Roman Period in Egypt.

## Historical background

Subsequent to the conquest of Egypt by Alexander the Great in 332 BC and the foundation of the new capital at Alexandria, a large number of Greeks immigrated to Egypt. During the Ptolemaic Period (332−30 BC) [15], people of Greek origin who settled in Alexandria largely cremated their deceased. However, those Greeks settling in other parts of the country tended to follow local customs and inhumed their deceased in wooden coffins with traditional Egyptian decoration. During the early Ptolemaic Period, indigenous Egyptians still chose traditional Egyptian burial equipment, such as shabtis, canopic jars, amulets, funerary papyri, etc. However, by the end of the Ptolemaic Period, use of these traditional grave goods had largely disappeared. Mummies were frequently decorated with masks, covered by panels of cartonnage or completely enclosed by cartonnage casing, and buried in traditional decorated coffins [35].

At the death of Cleopatra VII Philopator in 30 BC, Egypt became a province of the Roman state. During this period, the deceased were commonly buried in small graves and pits or in existing tombs, although some individual tomb architecture was still constructed [16]. Depending on the local landscape, older necropolises were expanded or new cemeteries were set up, usually near ancient sacred sites [17]. A frequently observed feature of Roman Period mummies was a portrait of the deceased, painted on a wooden panel or directly upon a linen shroud, in contrast to traditional Egyptian representations of the dead [2]. According to Riggs [17], incorporating portraiture on mummies was suggested as the most significant innovation originating from Greek and Roman artistic tradition influencing burial practices at that time. Even though inhumating deceased as mummies remained the preferred burial custom, mummies placed in coffins seemed to be rather the minority of what has been found. However, great attention was focused on the artistic decoration of the mummy, not only for aesthetic reasons but also to protect the body magically and ensure the rebirth of the dead in the afterlife [16]. The myth of the Egyptian god Osiris, who was installed as the ruler of the underworld [36] and assumed the role of the Great Judge, judging the souls of the deceased and shepherding them to immortality, was the central aspect of the Egyptian afterlife concept. With the establishment of the Ptolemies as regents of the Egyptian empire, the god Osiris was worshipped as the Greek god Sarapis. Subsequently, the cult of Sarapis was adopted by the Romans [37].

During the Graeco-Roman Period, mummies were kept at home and/or in a repository open to the public for some time in order to offer homage to the deceased before burial [2, 19, 38]. Intermittently, the mummies were brought together and buried in sepulchers or mass burials [19]. Placing a figurative representation of the deceased in the house of relatives was practiced during the pharaonic era (c. 3000−332 BC) [15] already [38]. Archaeological sites well-known for the discovery of Graeco-Roman mummies include Alexandria, el-Hibeh, Hermopolis, Akhmim (Panopolis), Antinoopolis, Thebes, the Fayoum Oasis, the Bahariya Oasis, the Dakhla Oasis and the Kharga Oasis.

## Material and methods

### Provenance, collection history, dating and previous investigations

**Fig 1:**
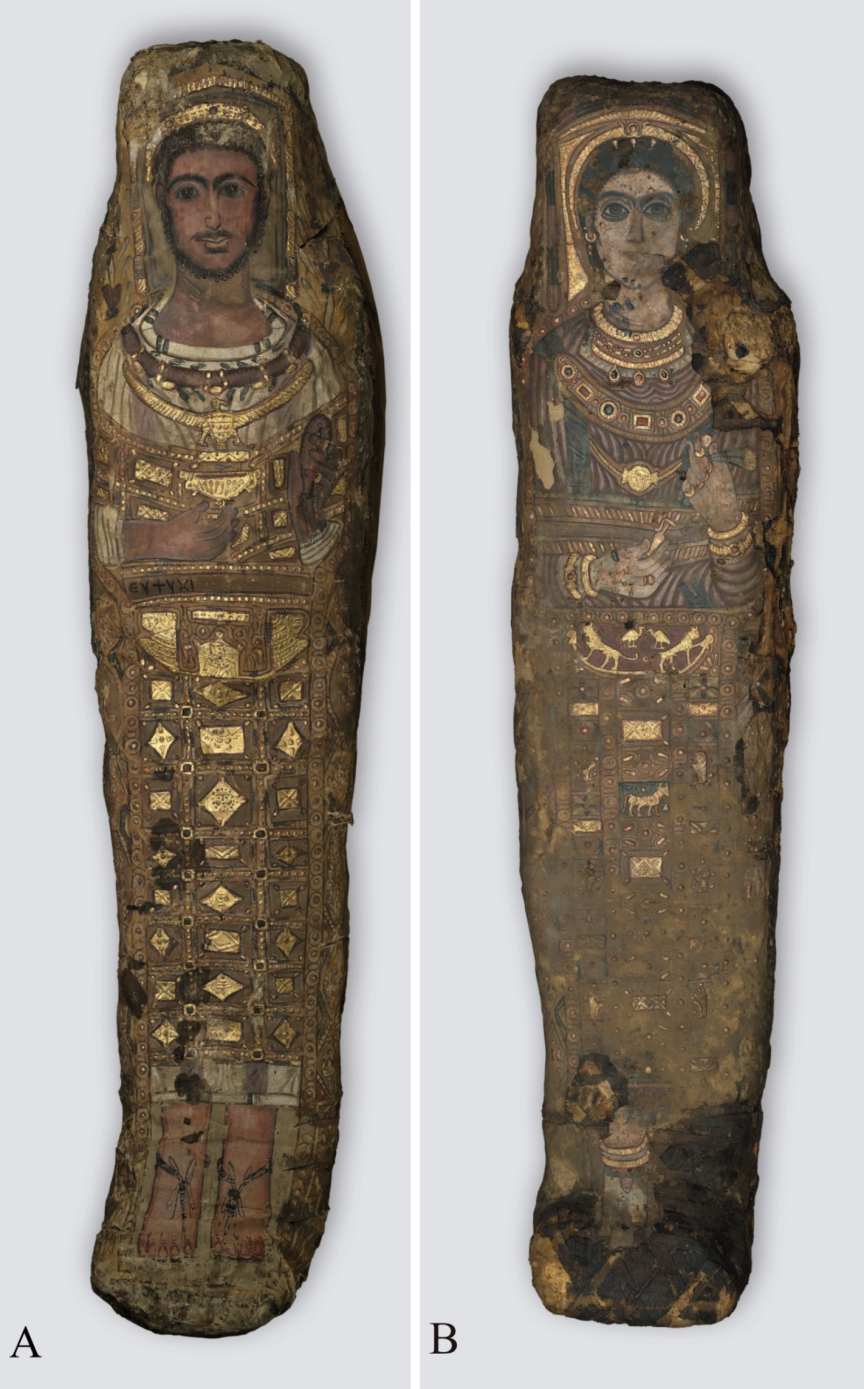
Stucco-shrouded portrait mummies housed at the Dresden State Art Collections. (A) male mummy (Aeg 777); (B) female mummy (Aeg 778) (© Sculpture Collection, Dresden State Art Collections, photos: H.-P. Klut/E. Estel).

Two of the mummies investigated are curated in the collection of antiquities (a part of the Sculpture Collection) in the Dresden State Art Collections in Dresden, Germany. The overall dimensions of the wrapped mummy of a male (Aeg 777) measure about 175 cm in length, with a maximum width of 40 cm and a maximum depth of 29.5 cm (Fig 1A). The overall dimensions of the wrapped mummy of a female (Aeg 778) measure about 164 cm in length, with a maximum width of 37.5 cm and a maximum depth of 29 cm (Fig 1B). The male’s shroud is better preserved than the female’s, which shows more damage, dirt and discolorations especially on the face, feet and sides of her shroud.

**Fig 2:**
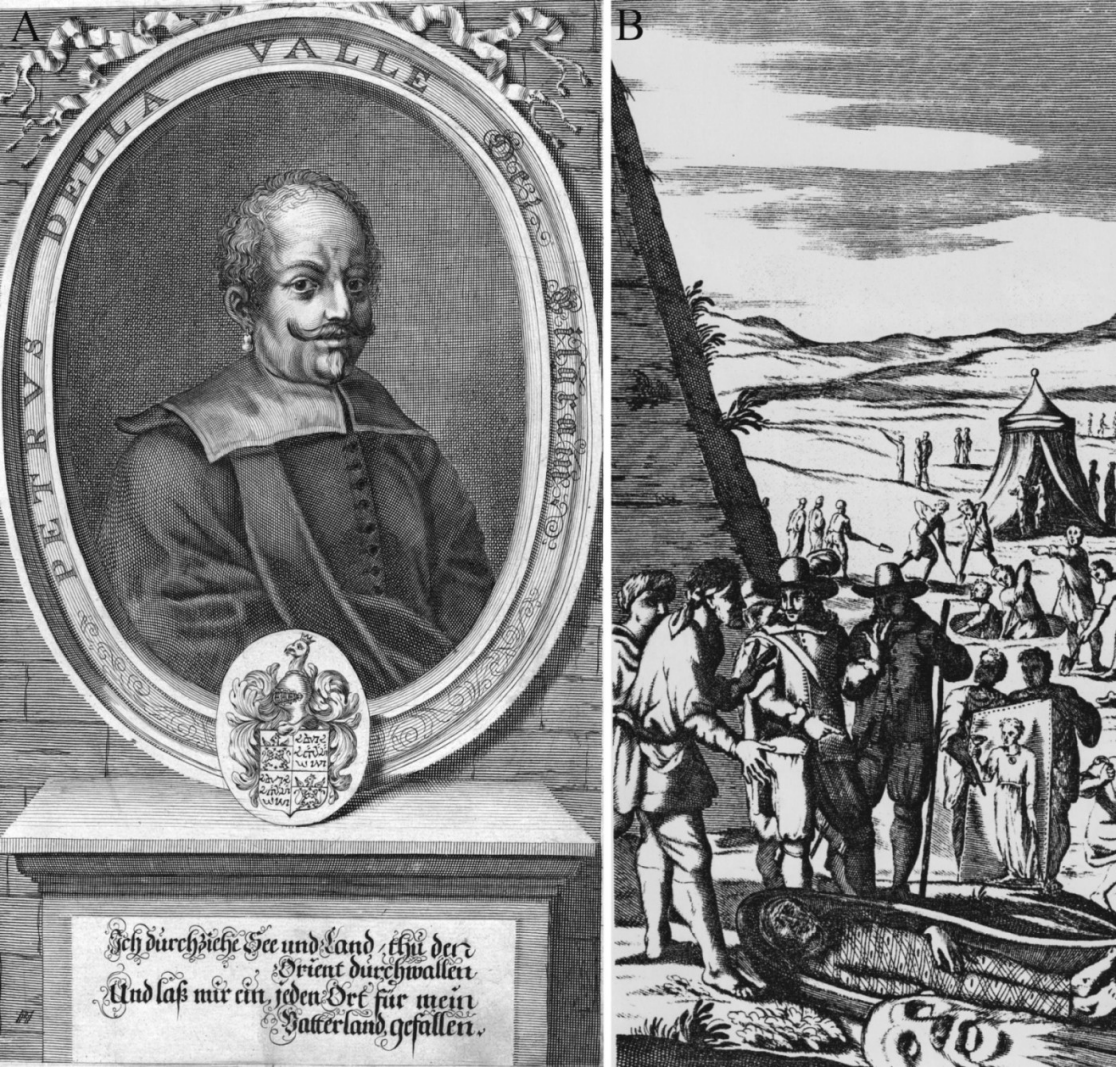
Reproductions of copper engravings. (A) a portrait of Pietro Della Valle; (B) the supposed moment of discovery of the mummies in Saqqara (adapted and reprinted under a CC BY license) [40].

In 1615, Pietro Della Valle (1586−1652) (Fig 2A) was on a pilgrimage through the orient when he acquired these two mummies following their discovery by local people in a rock cut tomb chamber in Saqqara (Fig 2B). He took them to Rome, where they became part of his collection of antiquities. In 1650, the first volume of his travelogue was published which included a report on the mummies’ discovery [39]. The German translation of this book contains a copper engraving capturing the supposed moment of discovery [40].

**Fig 3:**
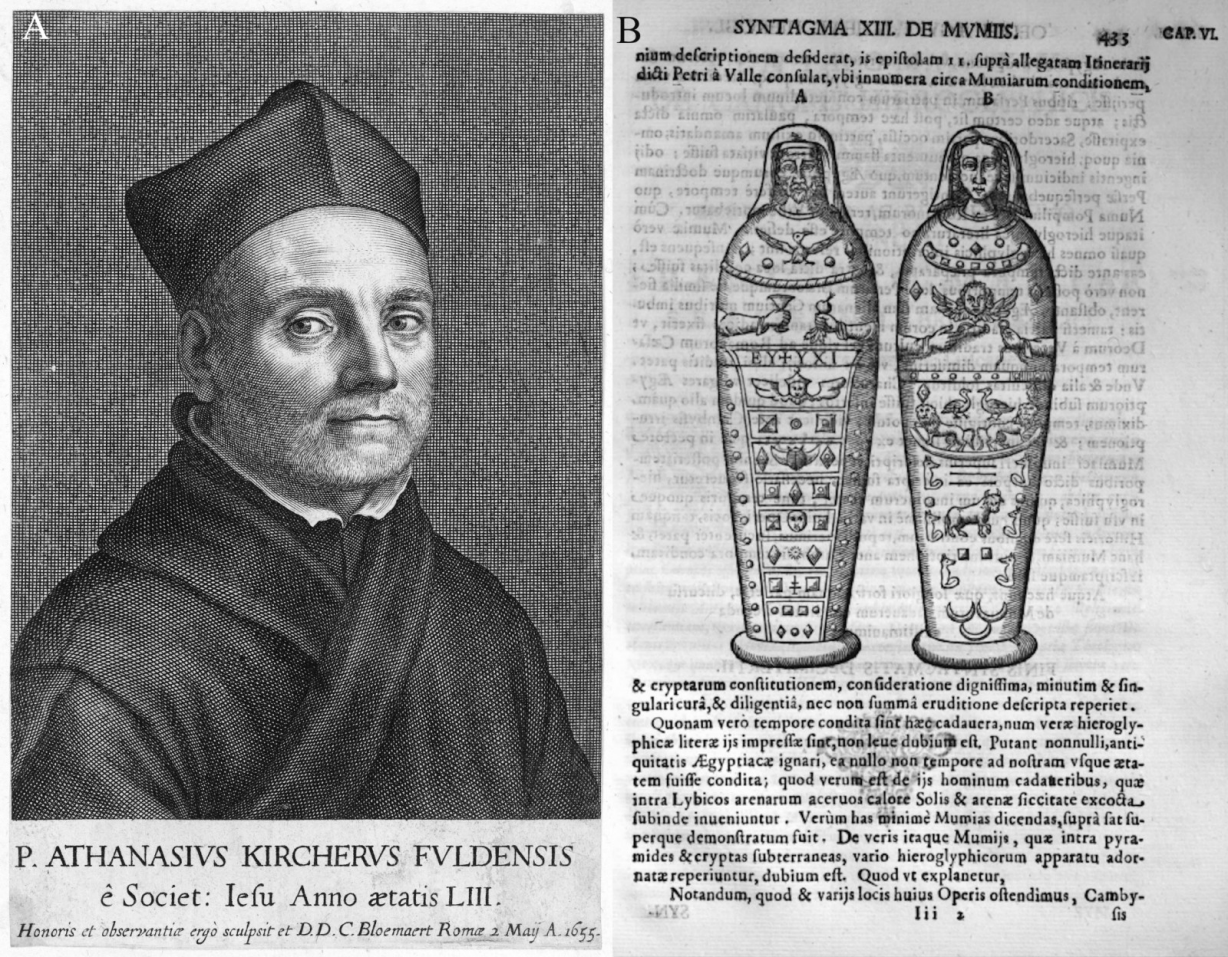
Reproductions of copper engravings. (A) a portrait of Athanasius Kircher made by Cornelis Bloemaert (original (inv. MP 12693) at Germanisches Nationalmuseum, Nuremberg, adapted and reprinted under a CC BY license); (B) the portrait mummies depicted by Athanasius Kircher (adapted and reprinted from [41] under a CC BY license).

In 1654, the Jesuit priest and scholar Athanasius Kircher (1602−1680) (Fig 3A) published two depictions of these two mummies in the third volume of *Oedipus Aegypticus* (Fig 3B) [41].

**Fig 4:**
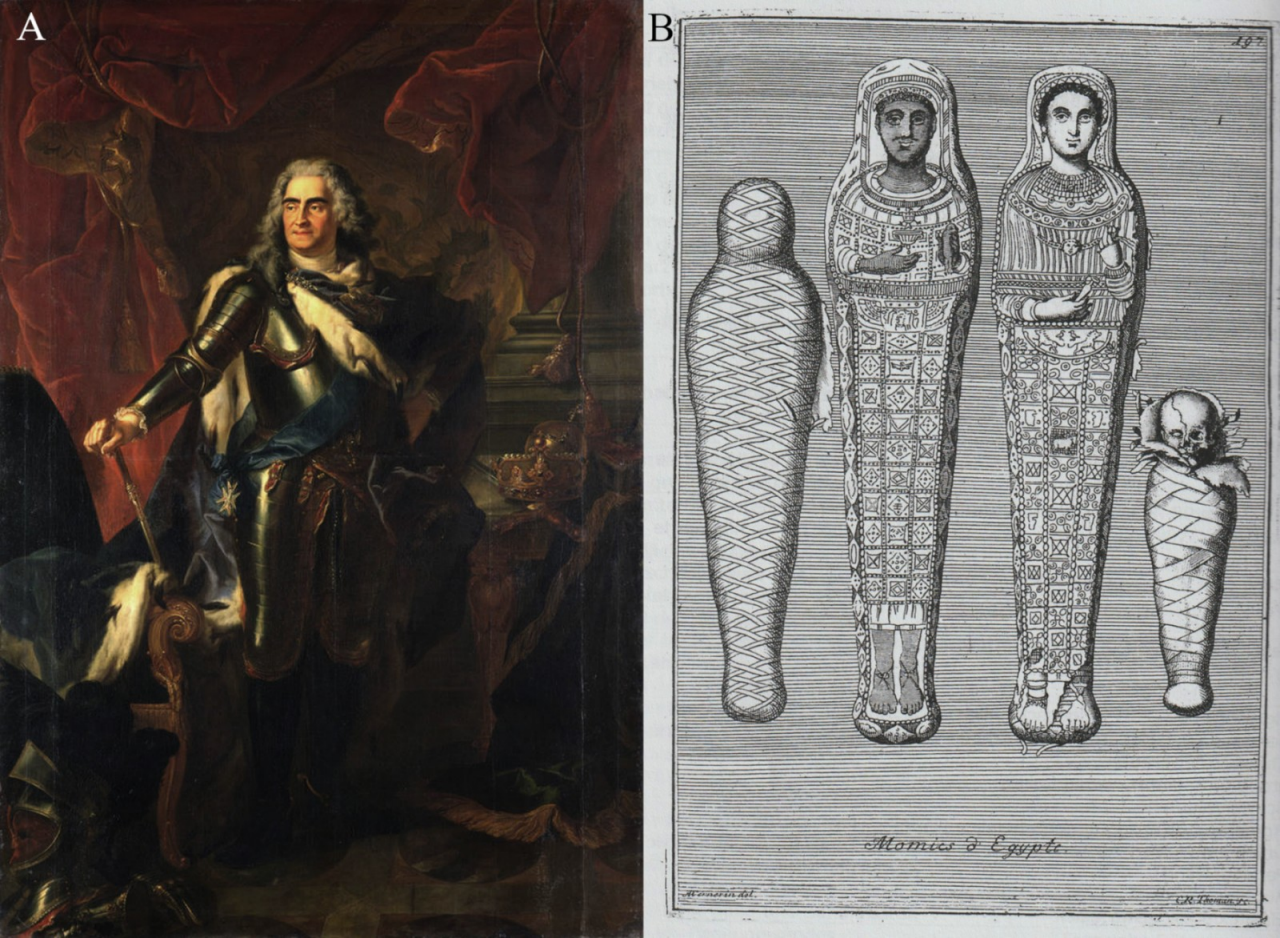
The mummies’ acquisition for the art collection of the Elector of Saxony and King of Poland August II. (A) a portrait of the sovereign made by Louis de Silvestre (original (inv. Gal.-Nr. 3943) at Old Masters Picture Gallery) (© Old Masters Picture Gallery, Dresden State Art Collections, photo: H.-P. Klut/E. Estel); (B) a copper engraving made by Anna Maria Werner and Christoph Raimund Thomann illustrating four mummies of the collection, including these two portrait mummies in the center (adapted and reprinted from [42] under a CC BY license).

The antiquities collection of Della Valle was sold after his death. These two mummies disappeared for decades later reappearing in the collection of Cardinal Filippo Antonio Gualtieri (1660−1728) at the beginning of the 18^th^ century. After Gualtieri’s death, his collection of antiquities was sold in Rome. In 1728, the mummies were acquired by Baron Raymond Leplat (1664−1742) to become part of the art collection of Friedrich August I, Elector of Saxony, alias August II, King of Poland, better known as August *the Strong* (1670−1733) (Fig 4A). In 1733, Leplat published a book on the sovereign’s art collection, containing a copper engraving illustrating these two mummies (Fig 4B) [42]. Further descriptions and illustrations were published by Johann G. Lipsius [43] and Wilhelm G. Becker [44].

In 1756, Johann J. Winckelmann (1717−1768) identified these two mummies as those described by Della Valle. He further determined the inscription on the male’s shroud as Greek language and dated the mummies to the Roman Period [45]. Nowadays, their time of origin is estimated between the late 3^rd^ to the middle of the 4^th^ century AD [46, 47], corresponding to the late Roman Period.

Unpublished reports from previous scientific investigations were made available to the authors by courtesy of the Dresden State Art Collections; they are listed in the supporting information (S1 Appendix) and summarized in the current paragraph. In 1987/1988, X-ray analysis was conducted at the hospital in Dresden-Friedrichstadt, supervised by Hans-Peter Pätzug; results were reported by Peter Begoff in 1988 and Wolfgang M. Pahl in 1990. Several conservation works have been performed on the Dresden mummies (most recently in 2018). In 2004, an analysis on the manufacturing of the shrouds and wrappings was done by Franziska Dötzel. A report on the mummies’ discovery, collection history and shroud decoration, as well as on shrouds with similar decoration, geographical and chronological provenance was written by Renate Germer in 2015.

**Fig 5:**
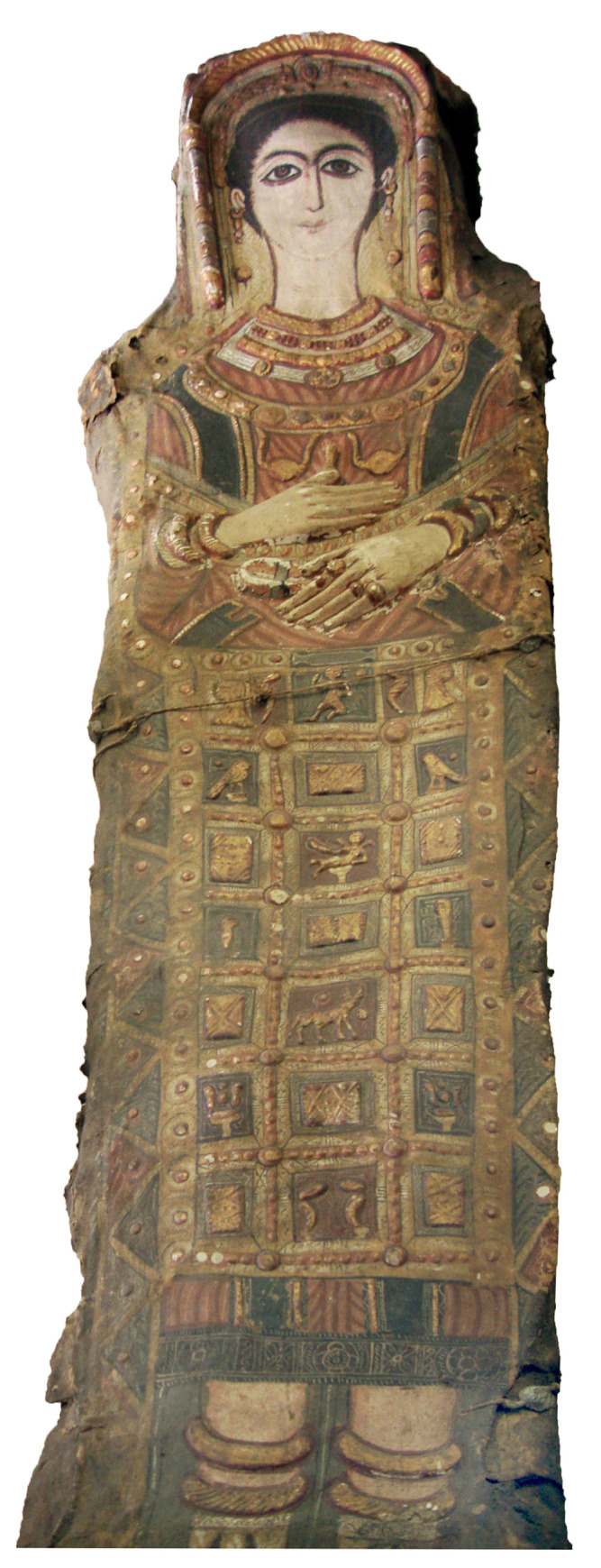
Female stucco-shrouded portrait mummy (CG 33281) housed at the Museum of Egyptian Antiquities, Cairo. © I. Badr.

The third mummy analyzed is that of a young female (CG 33281) with an overall length of 153 cm, maximum width of 35 cm and maximum depth 23 cm [33], housed at the Museum of Egyptian Antiquities, Cairo, Egypt (Fig 5). The mummy was discovered in the second half of the 19^th^ century, however, details about the circumstances of the mummy’s discovery are unknown. The mummy’s provenance is Saqqara [48, 49]. The specimen was first mentioned by Gaston Maspero who suggested the Byzantine Period (395−642 AD) as time of origin [48]. The first colored illustration was published by Albert Gayet [50]. The shroud with mummy portrait was described in detail as part of several publications [33, 49, 51]. Parlasca suggested a time of origin for the mummy as the middle of the 4^th^ century AD, based on a comparison of the portrait’s characteristics with the mummies in Dresden [51]. This time of origin was also reported in a work on portrait mummies by Corcoran [33].

### Palaeoradiological investigation

In 2010, a 6-slice CT system (Siemens Emotion 6, Florsheim, Germany) was used to scan 45 mummies in the Museum of Egyptian Antiquities, Cairo, within the framework of a scientific cooperation studying atherosclerosis in mummies from past societies [52]. Among this group of mummies was the portrait mummy of a young female (CG 33281), which was scanned in separate segments (head, thorax and abdomen, legs) with a slice thickness of 1.25 mm, a tube voltage of 130 kV, and varying tube currents (head with 116 mA, thorax and abdomen with 83 mA, legs with 100 mA). Images were reconstructed using various convolution kernels (head with B50s, thorax and abdomen with B30s, legs with B30s). In 2016, a 64-slice CT scanner (LightSpeed VCT, GE Medical Systems) was used at Städtisches Klinikum Dresden-Friedrichstadt, Radiologische Klinik to scan the portrait mummies housed at the Dresden State Art Collections. They were investigated with a slice thickness of 1.25 mm, a tube voltage of 120 kV, a tube current of 300 mA for the male and 575 mA for the female, and reconstructed with a standard convolution kernel.

Post processing and evaluation of the generated CT data was performed on several workstations with varying medical imaging software, such as OsiriX (3.7.1, 64-bit, Pixmeo SARL, Geneva, Switzerland) applied by the study’s first author. This included the generation of two-dimensional, multiplanar three-dimensional and volume rendered reconstructions. The radiodensity of foreign objects was assessed based on the Hounsfield Unit (HU) scale.

The authors obtained all necessary permits, which complied with all relevant regulations.

### Physical anthropological investigation

Soft tissue preservation was evaluated by applying the checklist of Panzer and colleagues [9]. Age at death was estimated based on several criteria which included the dental status with tooth development, tooth eruption [53] and dental wear [54]. Ectocranial suture closure was ascertained [55], however considered with caution due to artefacts which can be introduced by CT image reconstruction, as described in Barrett and Keat [56]. Markers of the musculoskeletal system relating to age, including the status of epiphyseal closure, trabecular bone density of long bones and evidence of degenerative changes, were evaluated as summarized in Ferembach and colleagues [57] and Scheuer and Black [58]. Sex was determined by assessing the shape and size of sex-specific morphological features of the skull and the pelvis, following Ferembach and colleagues [57]. Body height reconstruction was conducted by applying the methods from Pearson [59] and Trotter [60].

## Results

### Decoration on the mummy shrouds

The shrouds show whole-body representations of the deceased combined with various decorative elements and religious motifs partially made of stucco, painted and partially gilded. The deceased wear a garment, usually identified as a Greek *chiton* or a Roman tunic, but with sleeves. The male (Aeg 777) wears a white tunic showing two vertical mauve textile stripes, so-called *clavi* (decorative elements of a Roman tunic), extending from the shoulders to the ankles. There are eight thin olive-colored stripes arranged in pairs around the neckline of the white tunic (Fig 1A). He wears sandals with one single strap between the first and second toes. The female (Aeg 778) is clad in a reddish tunic including two olive-colored *clavi* extending from the shoulders. The representations of footwear are not well preserved (Fig 1B). The copper engraving illustrating the mummies’ status of preservation in 1733 (Fig 4B) [42] suggests that the female either was depicted barefoot or wore sandals similar to the male. However, the preserved remnants of decoration, including a shoelace, objects in the shape of pennants and the gray (not nude) color of the foot area (Fig 1B) rather indicate closed shoes, similar to those visible on the young female (CG 33281) described below.

The shrouds illustrate covers extending from the waist to the ankles, indicating stylized representations of bead-nets (Fig 1). They show vertical rows of square panels containing various decorative elements, such as rhomboid, rectangular and square items (Aeg 777), floral patterns, figurative motifs and animals (Aeg 778). The bead-nets show one large figurative motif visible directly below the arms, identified as a stylized winged scarab (Aeg 777) and as a golden barque (boat) with small human heads in profile on bow and stern, lions in antithetic position in the center and two birds representing vultures above the lions’ backs (Aeg 778). The female’s illustration of the bead-net further shows the Apis bull with a very small golden sun disk above his back. The vertical rows include small depictions of three of the four Sons of Horus in squatting posture on each side. The lowest panel, which is expected to visualize the fourth Son of Horus, is poorly preserved.

**Fig 6:**
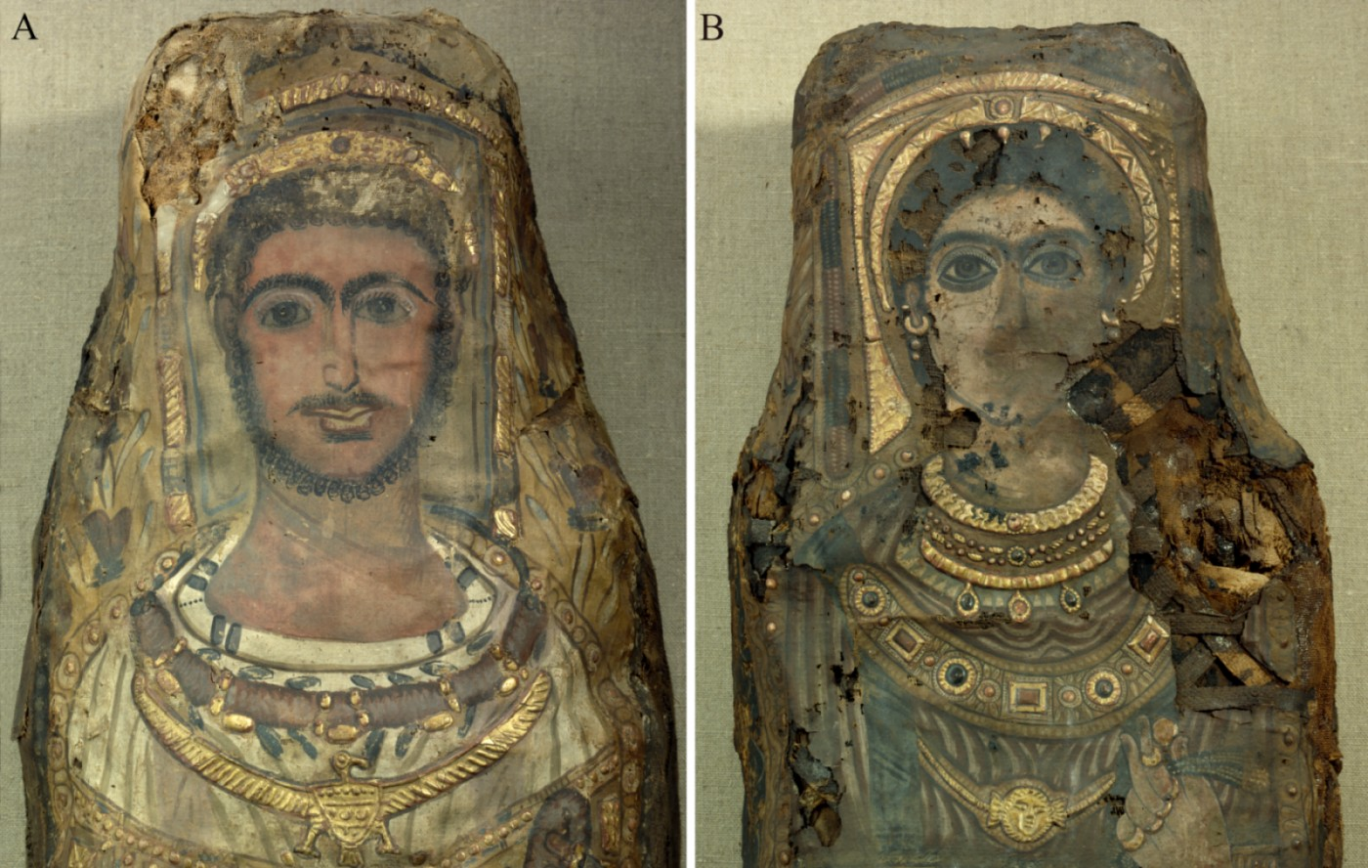
Detail views of the mummy portraits and jewelry depicted on the shrouds. (A) male mummy (Aeg 777); (B) female mummy (Aeg 778) (© Sculpture Collection, Dresden State Art Collections, photos: H.-P. Klut/E. Estel).

The mummies’ heads are enclosed by partially gilded stucco frames (Fig 6). The portraits include a tempera painted face and Roman hairstyle crowned with a golden wreath. The male portrait (Aeg 777) depicts the face of an adult with dark short curls, a moustache, a chin beard, and prominent eyebrows. A remarkable garland made of red rose blossoms, green leaves and golden beads is placed around his neck. A golden depiction of the Egyptian vulture goddess Nekhbet is on his breast. The partially preserved female portrait (Aeg 778) shows dark hair, three golden hairpins, and prominent eyebrows. Her golden wreath includes a small sun disk in the center flanked by cobras or cow horns, probably symbolizing the crown of the Egyptian goddess Hathor-Isis. Her jewelry comprises earrings, a linked necklace of minted gold foil, several golden necklaces with precious stones, two golden bracelets on each wrist and two bracelets on the right ankle above the cuff of her shoe. A large decorative band or pectoral with various colored precious stones and a golden necklace with a circular pendant showing facial features of a Gorgon's head are visible on her breast.

**Fig 7:**
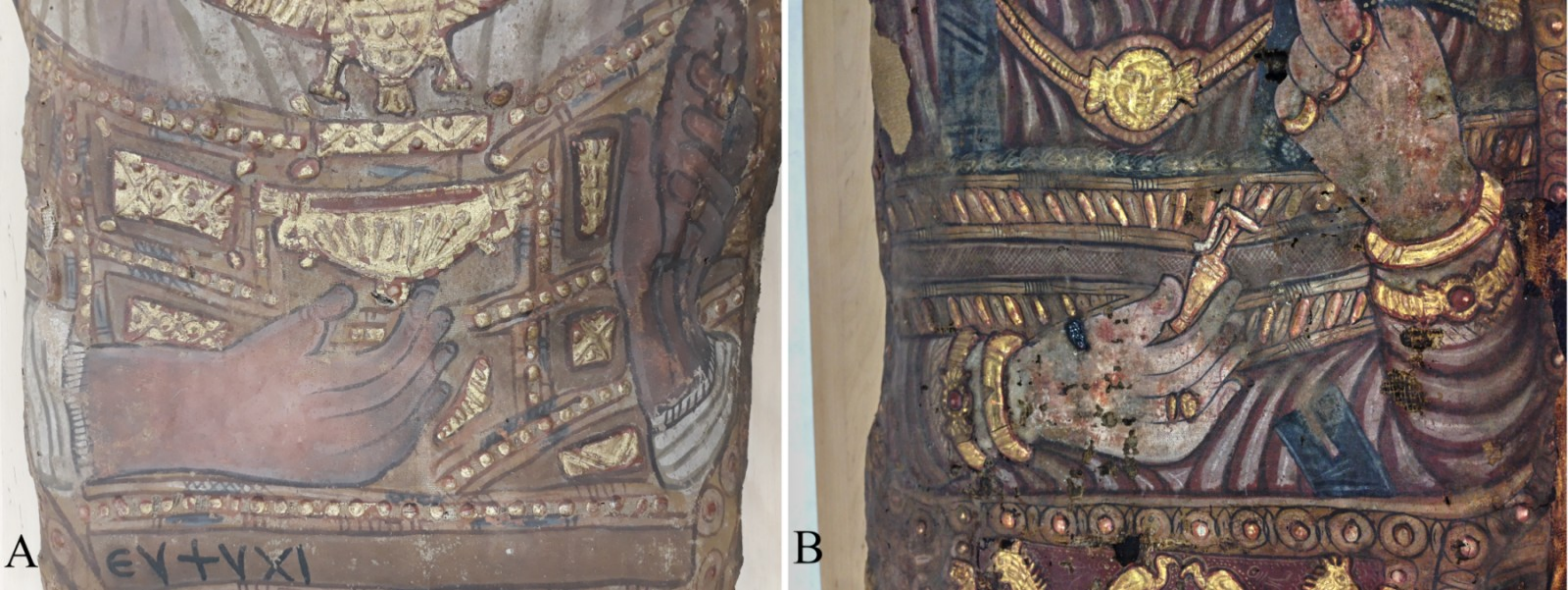
Detail views of the objects visible in the hands. (A) male (Aeg 777) with a so-called *kantharos* in his right hand and a garland or a folded wreath of blossoms in his left hand. Note also the Greek inscription below his right forearm; (B) female (Aeg 778) with a so-called *lekythos* in her right hand. (© Sculpture Collection, Dresden State Art Collections, photos: M. Gander/M. Loth).

The male (Aeg 777) carries a golden cup, so-called *kantharos* (a Greek vessel usually filled with wine), in his right hand and a garland or a folded wreath of blossoms in his left hand that is further adorned with one gold ring on the forefinger and another one on the little finger (Fig 7A). The Greek inscription ΕΥΨΥΧΙ (Eupsychi) below his right arm is translated as “Farewell”. The female (Aeg 778) carries a golden vessel with a handle, so-called *lekythos* (a Greek vessel usually filled with oil), in her right hand whose ring finger is decorated with two rings (Fig 7B). She carries two assumed ears of corn and one small circular golden item, probably a stylized representation of a poppy seed-pod, in her left hand which is adorned with five rings (Fig 6B).

**Fig 8:**
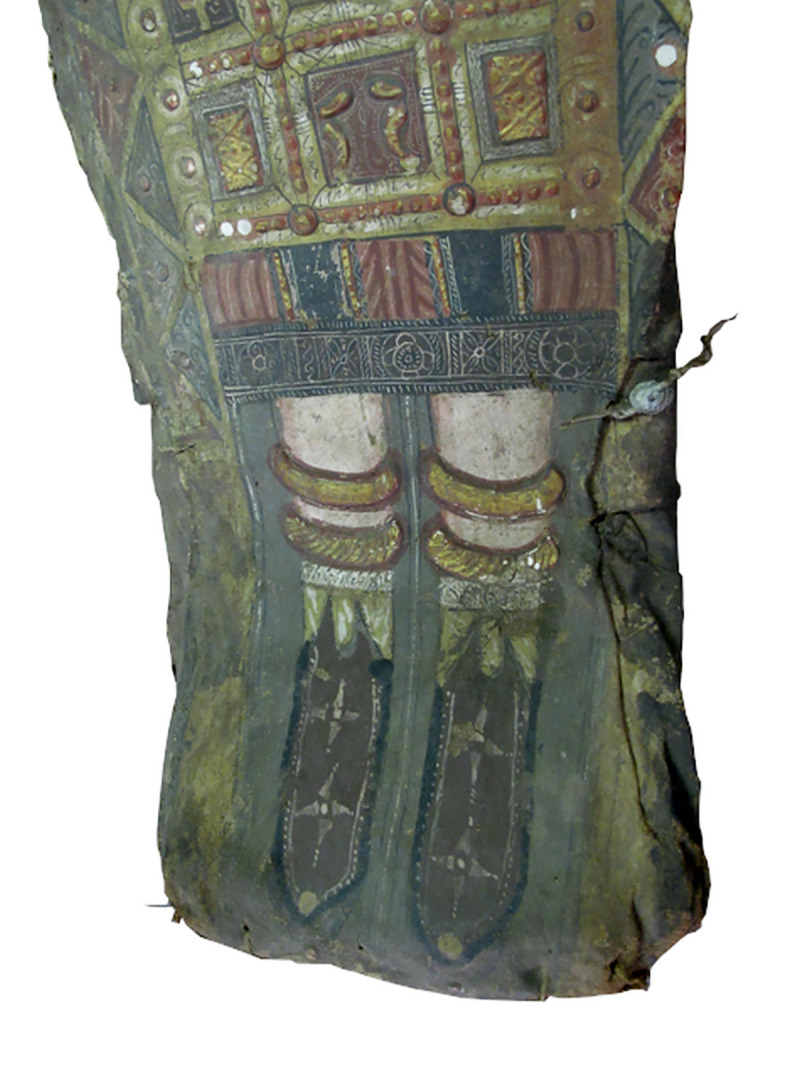
Young female mummy (CG 33281). The detail photo focuses on the red-brown pointed shoes, surrounded by white seams on the front and sides, decorated with a circular object on each tip and two white stars in the center of each shoe (© I. Badr).

The young female (CG 33281) is dressed in a red-colored tunic with sleeves and olive-colored *clavi* (Fig 5). She wears red-brown pointed shoes with white seams on the front and sides, a small circular yellow object on each tip and two white stars in the center of each shoe (Fig 8).

The lower part of the body, from the level of the waist to the ankles, is covered by a stylized representation of a bead-net (Fig 5). The stylized bead-net forms a grid pattern with seven rows of square panels; the top row contains five panels and the remaining rows each contain three panels. Within these panels various ornaments, symbols and divine beings in gilded raised relief are visible. These depictions include ancient Egyptian motifs also occurring on the Dresden mummies, such as pairs of upraised cobras representing the uraeus goddess, the Apis bull and the human heads in profile with head coverings resembling a pharaoh. Additional elements following Egyptian conventions are visible, such as falcons, standing mummiform figures and a design that may be identified as crowns with sun-disk and cow horns or cobras. Two depictions derive from Graeco-Roman art; a naked human figure is shown in the stance of a fighter using a spear or harpoon (not present) in the top center panel, identified as the Egyptian god Harpocrates by Edgar [49] and Corcoran [33] and a hovering human figure with wings holding an object in his right hand suggestive of a wreath or crown in the third row, center panel. This divine being could represent putto, cupid or winged genius.

**Fig 9:**
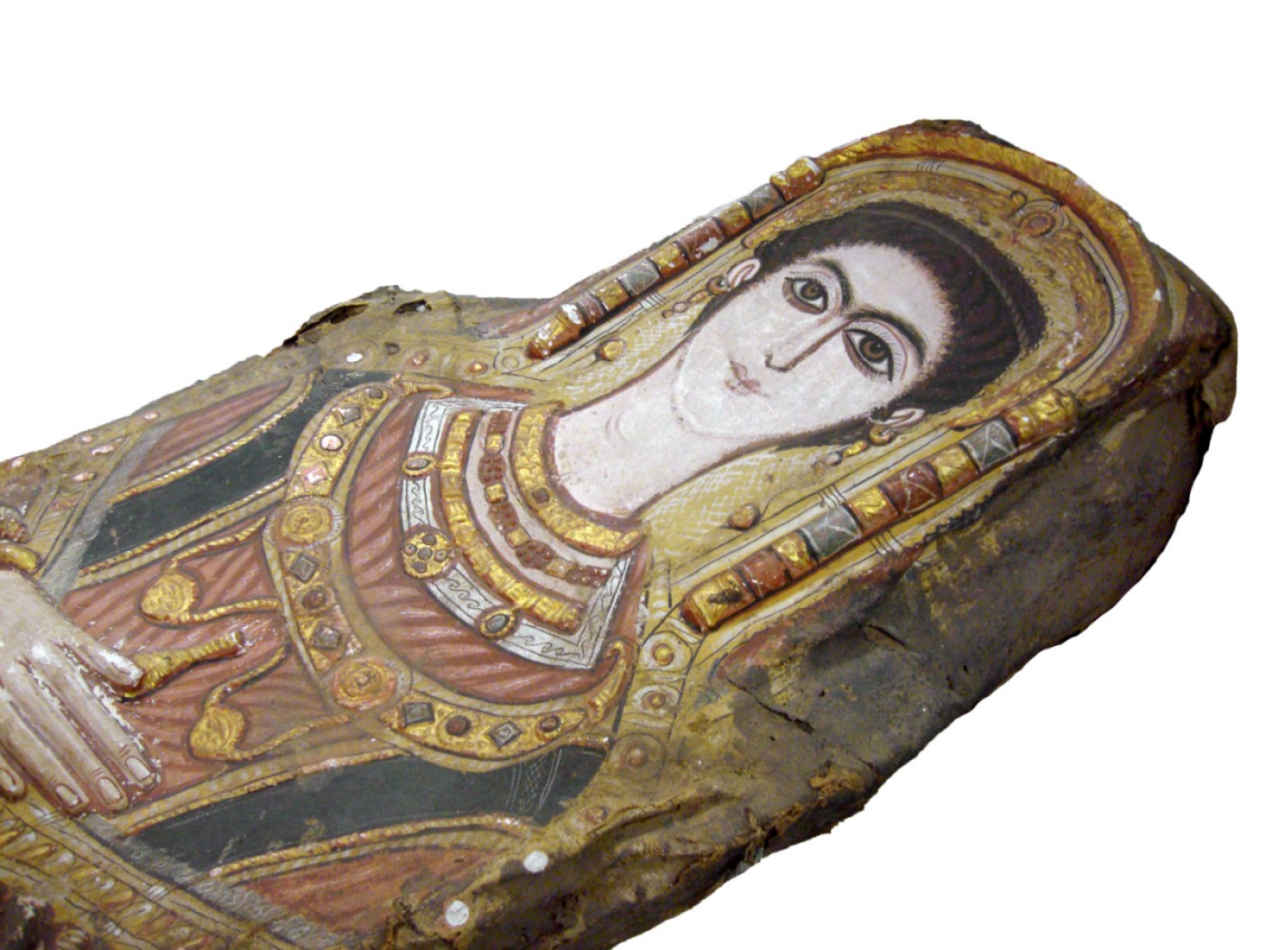
Young female mummy (CG 33281). The photo shows the decoration of the mummy shroud, focusing on the portrait and torso (© I. Badr).

The portrait of the young female (CG 33281) is almost perfectly preserved (Fig 9). There are stylistic differences to the female mummy in Dresden, such as a more oval form of the face, eyes less wide open and a narrower nose. The head is encircled by a golden wreath with a sun-disk in its center protected by cobras, similar to the depiction of the female mummy in Dresden. Three raised stucco dots depicted above the head might be interpreted as heads of hairpins. Jewelry consists of earrings, three golden necklaces, two golden bracelets on each wrist and each ankle, one additional blue bracelet on the left wrist and four finger rings on the left hand. Two golden medallions depicting faces, probably the aegis with Gorgon’s head, are suspended from a golden band or pectoral on her breast, which contains a row of gems of different colours. The right hand holds a conical vessel without handle, probably used for oil, and the left hand holds a wreath. As this mummy shows many similarities in technique, style and iconography compared to the Dresden mummies, it can be concluded, that it was produced during the same time period and in the same workshop. The high-quality of these three mummy shrouds indicate an upper socioeconomic status of the deceased.

### Artificial mummification and preservation

**Fig 10:**
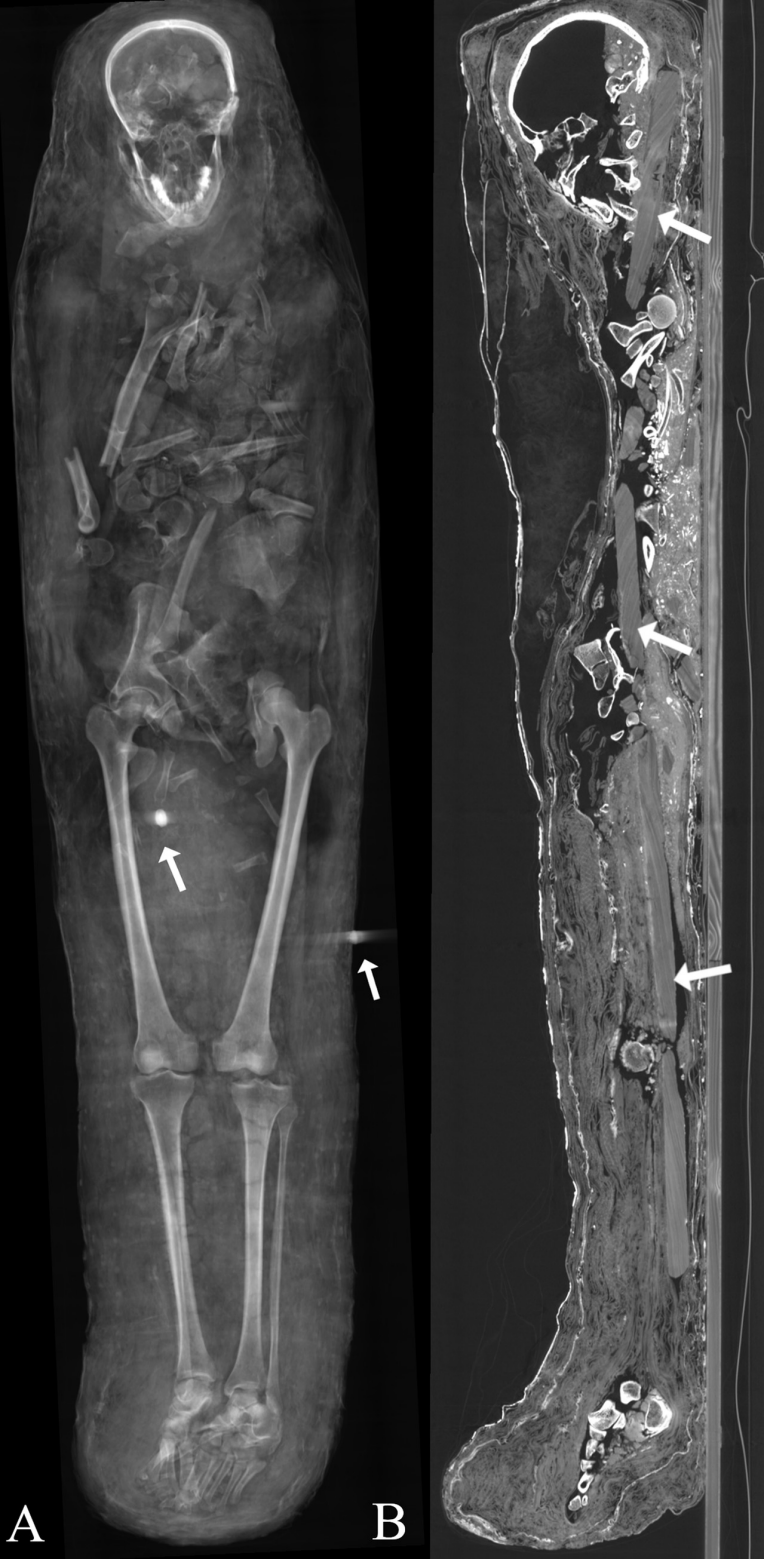
Male mummy (Aeg 777). (A) thick-slab mean intensity coronal multi-planar reconstruction illustrates one metal object close to the right femur, suggested to be a translocated metal seal, and another metal seal on the outer textile layer (arrows); (B) sagittal multi-planar reconstruction visualizes parts of a broken wooden board placed beneath the body (arrows).

The male’s body (Aeg 777) in supine position shows no perforation of the ethmoid bone and/or the sphenoid bone that would indicate a transnasal brain removal (Fig 10). Brain remnants are not preserved. A conglomerate of two vertebrae, small size bone fragments and sediment is visible inside the skull cavity, most probably displaced into the skull as a result of extensive damage to the basioccipital and occipitocervical regions of the mummy. The body is covered by many textile layers of varying density, showing a lower density in the upper body region.

**Fig 11:**
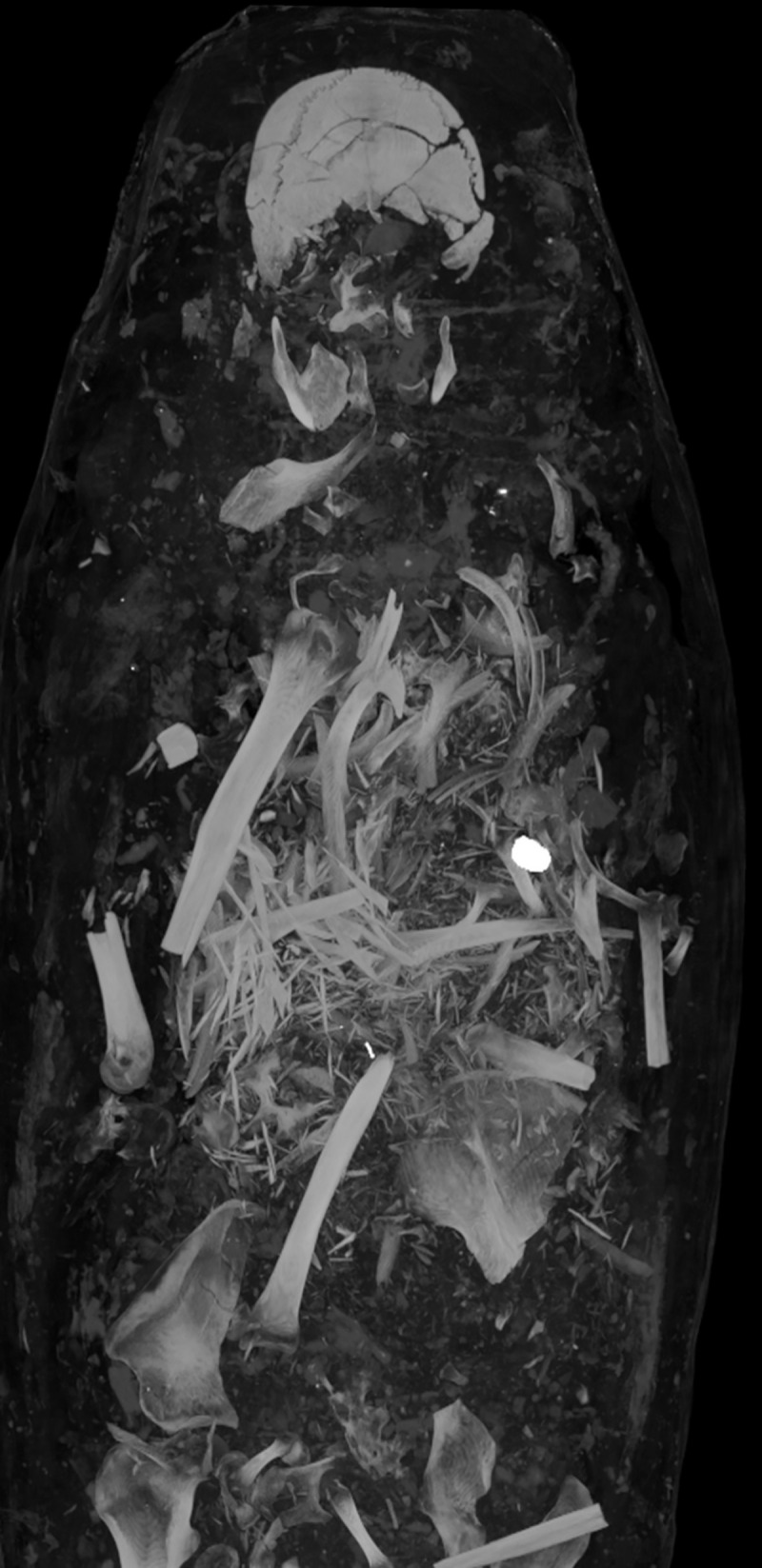
Male mummy (Aeg 777). Thick-slab maximum intensity coronal multi-planar reconstruction illustrates disarticulated and partially broken bones of the trunk and the upper extremities, as well as a foreign object in the upper left thoracic region suggested to be a translocated metal seal. Scattered radiopaque material (possibly sediment and plant materials) are visible.

Skeletal elements of the upper body are broadly disarticulated, dislocated and broken (Fig 11). The shape of the bony margins indicate the breaks most likely occurred during post mortem manipulation of the body. Internal organs are not preserved. Bone fragments of various sizes and scattered radiopaque material (possibly sediment and plant material) are visible throughout the upper body.

**Fig 12:**
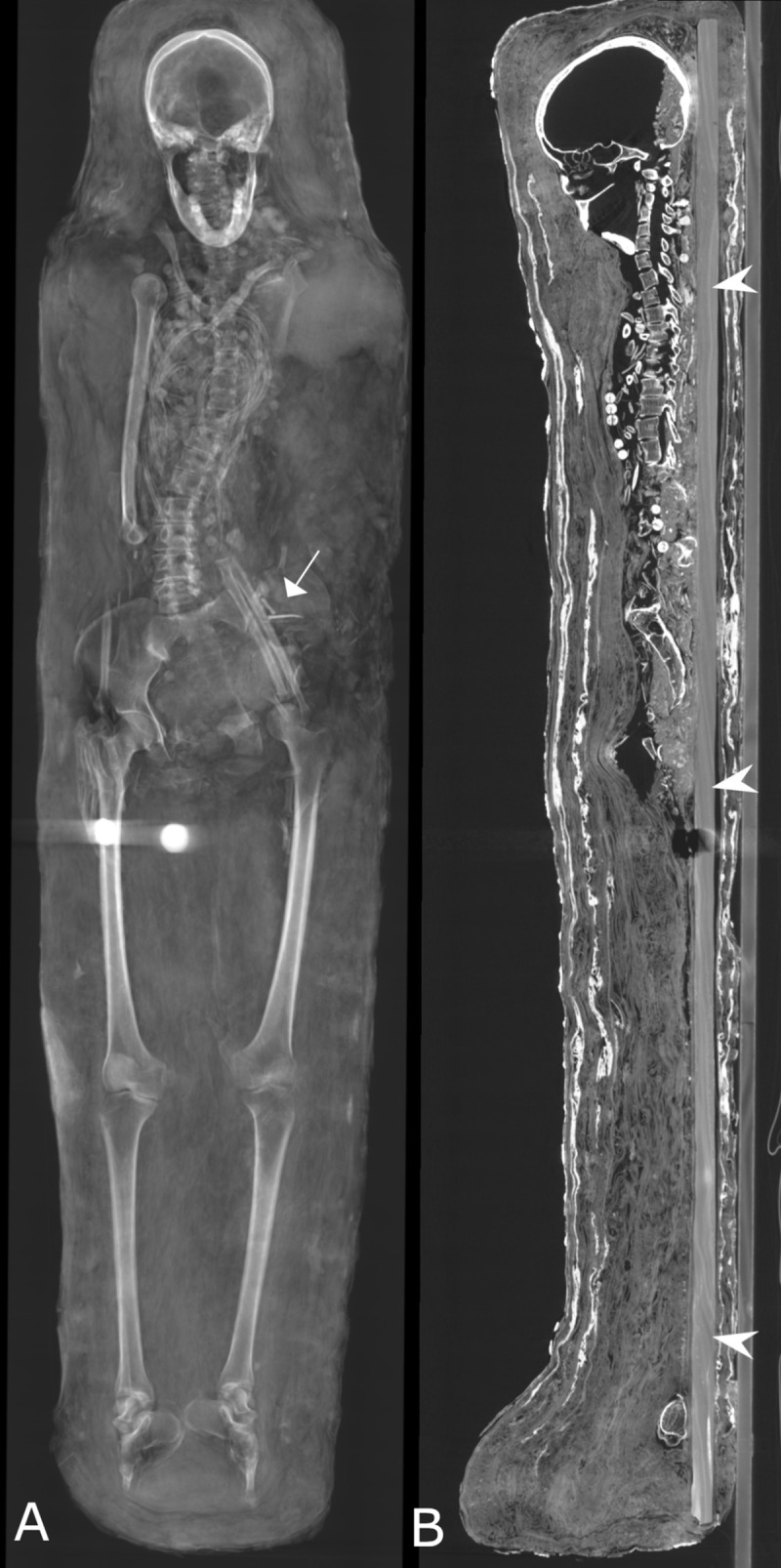
Female mummy (Aeg 778). (A) thick-slab mean intensity coronal multi-planar reconstruction visualizes two circular metal dense foreign objects close to the right femur, probably representing coins or medallions. Two nails were identified in the abdominal region (arrow); (B) sagittal multi-planar reconstruction shows an intact wooden board identified beneath the body (arrow heads).

The female’s body (Aeg 778) is arranged in supine position (Fig 12). It is assumed that the arms were originally placed alongside the body, because hand bones were identified lateral to the femoral heads. Manipulation of the ethmoid bone and/or the sphenoid bone was not detected. Remnants of brain and internal organs are not preserved. A radiopaque conglomerate of small bone fragments and sediment inside the skull cavity, and a similar conglomerate near the occiput and the C2 cervical vertebra as well as inside the spinal canal most probably occurred as a consequence of craniocervical disarticulation. Skeletal elements of the upper body and upper extremities are also broadly disarticulated, dislocated and partially broken, although to a lesser extent than those of the male. The mummy is covered by many textile layers of rather uniform radiodensity.

**Fig 13:**
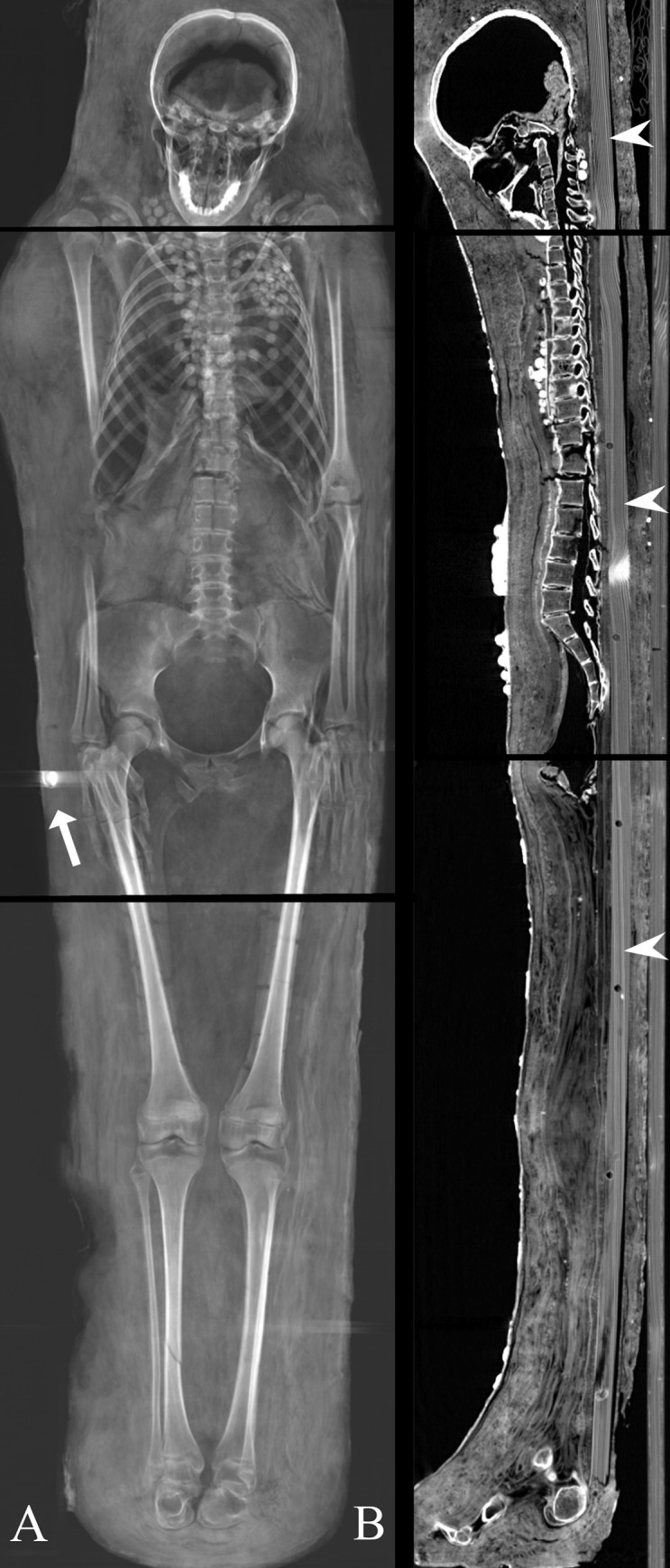
Young female mummy (CG 33281). (A) thick-slab mean intensity coronal multi-planar reconstruction illustrates one of seven metal seals fixed on the outermost textile layer (arrow); (B) sagittal multi-planar reconstruction shows an intact wooden board placed beneath the body inside the wrappings (arrow heads).

The mummy of the young female (CG 33281) is arranged in supine position with the arms lying alongside the body and the hands resting flatly on the thighs (Fig 13). Some skeletal elements are fractured and dislocated. The upper body is quite flat.

**Fig 14:**
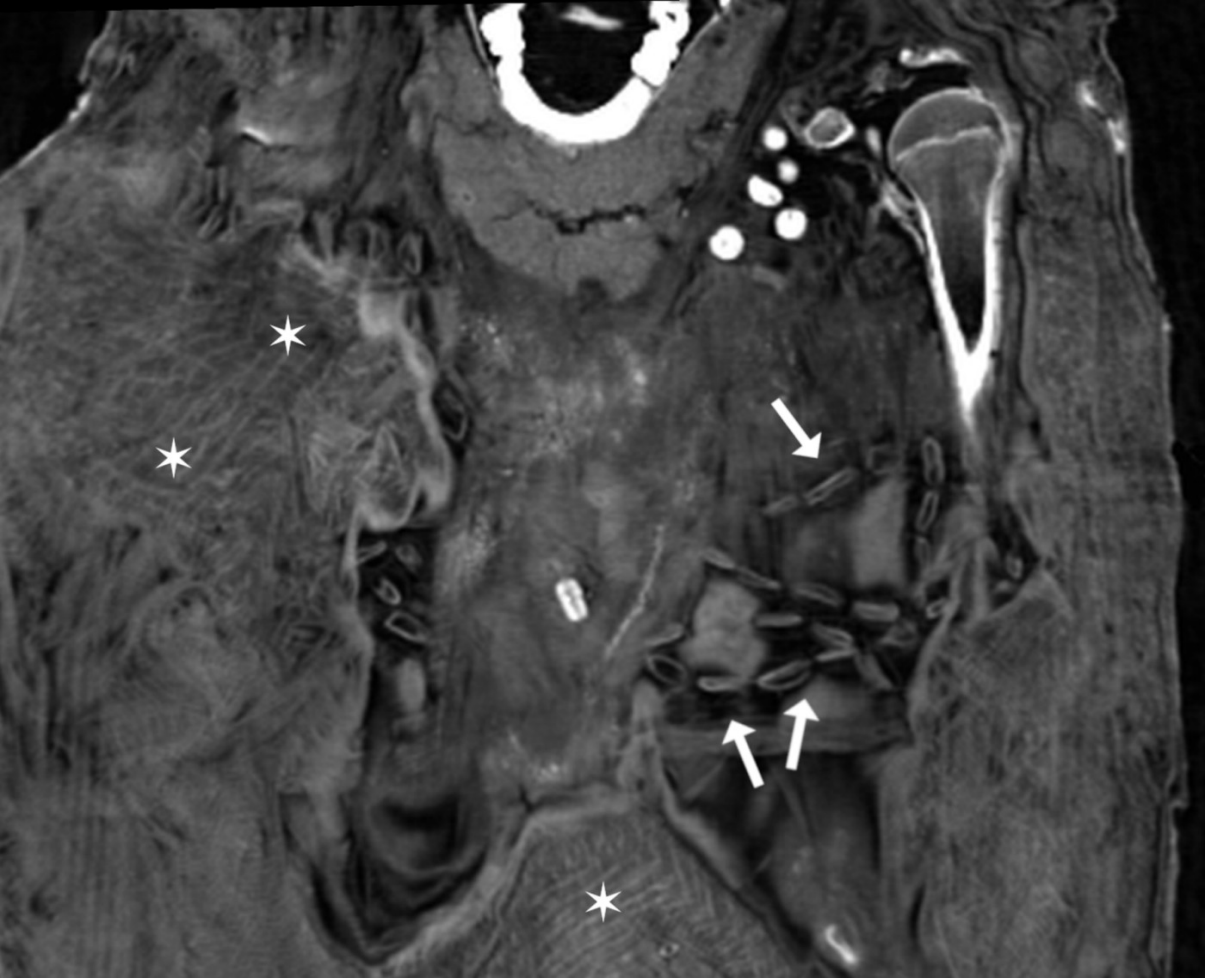
Young female mummy (CG 33281). Coronal multi-planar reconstruction depicts several segments of the innermost textile layer with a geometric pattern (asterisks) and longitudinal thin-walled low density beads in the thoracic region (arrows).

The body is wrapped tightly and densely by numerous textile layers with the innermost layer showing a geometric pattern (Fig 14).

**Fig 15:**
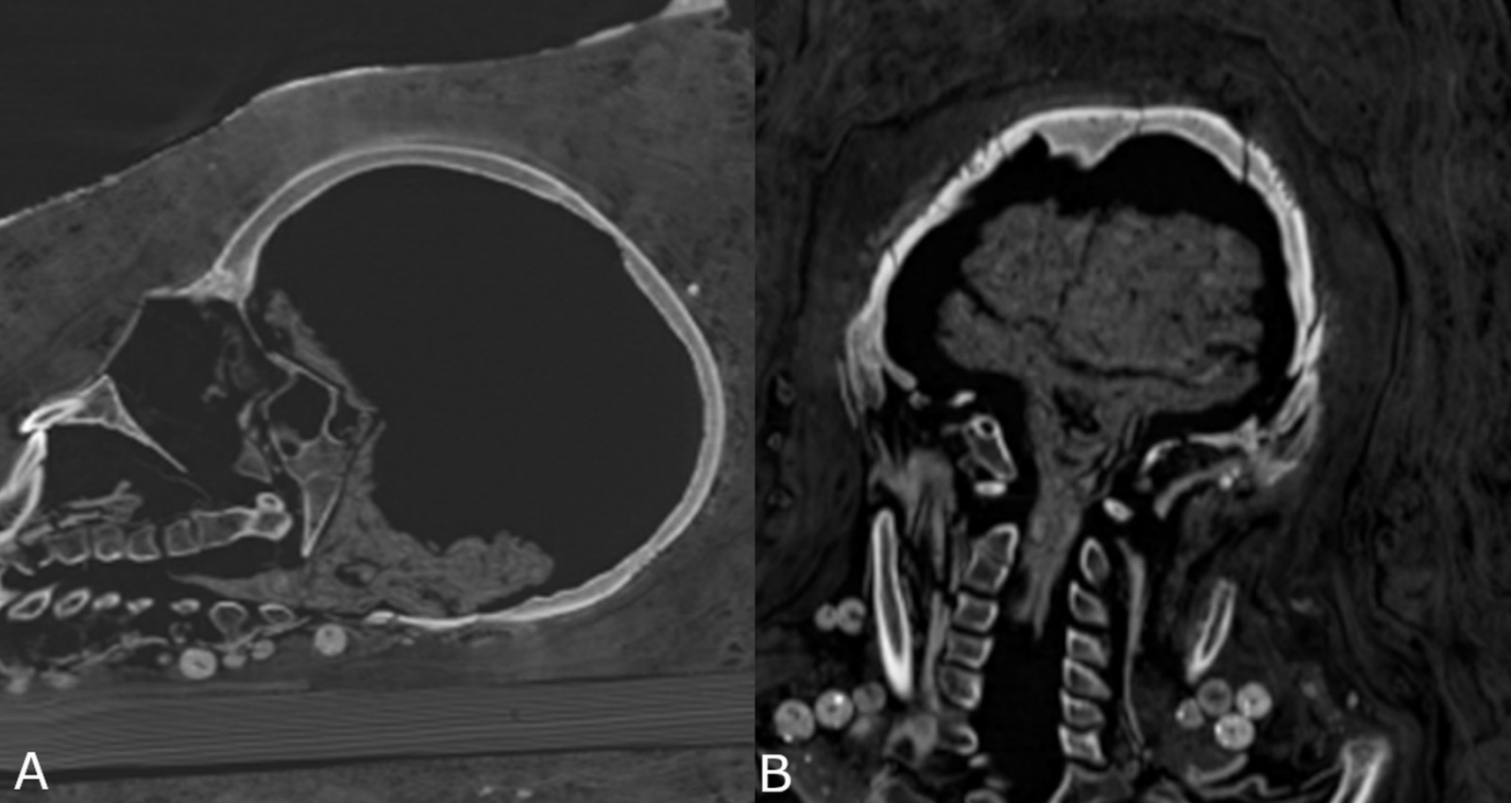
Young female mummy (CG 33281) with shrunken brain and brainstem preserved. (A) sagittal multi-planar reconstruction; (B) coronal multi-planar reconstruction.

Remnants of internal organs were identified. The preservation of the shrunken brain is good, allowing the identification of the cerebrum, cerebellum and brainstem (Fig 15).

The results of detailed evaluation of the mummies’ soft tissue preservation by applying the checklist published by Panzer and colleagues [9] are given in Table 1. The total scores yielded values of 9.0 for the male (Aeg 777), 58.0 for the female (Aeg 778) and 114.0 for the young female (CG 33281). Considering a possible maximum score of 200.0 per specimen, the values indicate poor soft tissue preservation of the male (Aeg 777) and a slightly better preservation of the female (Aeg 778), especially of the head and the musculoskeletal system. In contrast, the soft tissues of the young female (CG 33281) are significantly better preserved.

**Table 1:** Results on the checklist and calculated scores for the soft tissue preservation.

Mummy	Male mummy (Aeg 777)		Female mummy (Aeg 778)		Young female mummy (CG 33281)	
Checkpoints	-/+	Score	-/+	Score	-/+	Score
**A. Soft tissues of the head and musculoskeletal system**		**9.0**		**55.0**		**87.0**
**A.1. Head**		**1.0**		**16.0**		**16.0**
*nose*	-	(4)	+	4	+	4
*auricle* right	-	(2)	+	2	+	2
*auricle* left	-	(2)	+	2	+	2
*ossicles* right	+	1	+	1	+	1
*ossicles* left	-	(1)	+	1	+	1
*bulb and/or lens* right	-	(1)	+	1	+	1
*optic nerve* right	-	(1)	+	1	+	1
*eye muscles* right	-	(1)	+	1	+	1
*bulb and/or lens* left	-	(1)	+	1	+	1
*optic nerve* left	-	(1)	+	1	+	1
*eye muscles* left	-	(1)	+	1	+	1
*falx*	-	(2)	-	(2)	-	(2)
*tentorium*	-	(2)	-	(2)	-	(2)
**A.2. Musculoskeletal system**		**8.0**		**39.0**		**71.0**
**A.2.1. Tendons and/or musculature**		**8.0**		**22.0**		**40.0**
Neck and trunk						
*skull-base*	-	(4)	+	4	+	4
*neck*	-	(4)	+	4	+	4
*thoracic and/or lumbar spine*	-	(4)	-	(4)	+	4
*pelvis*	-	(4)	-	(4)	+	4
Extremities						
*upper arm* right	-	(2)	-	(2)	+	2
*upper arm* left	-	(2)	-	(2)	+	2
*forearm* right	-	(2)	-	(2)	+	2
*forearm* left	-	(2)	-	(2)	+	2
*hand* right	-	(2)	+	2	+	2
*hand* left	-	(2)	-	(2)	+	2
*thigh* right	-	(2)	+	2	+	2
*thigh* left	-	(2)	+	2	+	2
*lower leg* right	+	2	+	2	+	2
*lower leg* left	+	2	+	2	+	2
*foot* right	+	2	+	2	+	2
*foot* left	+	2	+	2	+	2
**A.2.2. Peri- and intra-articular soft tissues**		**0.0**		**9.0**		**23.0**
shoulder right *rotator cuff*	-	(2)	-	(2)	+	2
shoulder right *long biceps tendon*	-	(2)	-	(2)	-	(2)
shoulder right *capsule and/or labrum*	-	(2)	-	(2)	-	(2)
shoulder left *rotator cuff*	-	(2)	-	(2)	+	2
shoulder left *long biceps tendon*	-	(2)	-	(2)	-	(2)
shoulder left *capsule and/or labrum*	-	(2)	-	(2)	+	2
hip right *capsule and/or labrum*	-	(4)	-	(4)	+	4
hip left *capsule and/or labrum*	-	(4)	-	(4)	+	4
knee right *anterior cruciate ligament*	-	(1.5)	+	1.5	+	1.5
knee right *posterior cruciate ligament*	-	(1.5)	-	(1.5)	+	1.5
knee right *medial meniscus*	-	(1.5)	+	1.5	+	1.5
knee right *lateral meniscus*	-	(1.5)	+	1.5	+	1.5
knee left *anterior cruciate ligament*	-	(1.5)	+	1.5	+	1.5
knee left *posterior cruciate ligament*	-	(1.5)	-	(1.5)	+	1.5
knee left *medial meniscus*	-	(1.5)	+	1.5	-	(1.5)
knee left *lateral meniscus*	-	(1.5)	+	1.5	-	(1.5)
**A.2.3. Intervertebral discs**		**0.0**		**8.0**		**8.0**
*thoracic spine*	-	(4)	+	4	+	4
*lumbar spine*	-	(4)	+	4	+	4
**B. Organs and organ systems**		**0.0**		**3.0**		**27.0**
**B.1. Central nervous system and peripheral nerves**		**0.0**		**3.0**		**9.0**
brain *mass/fragments*	-	(1)	-	(1)	-	(1)
**or**						
brain *cerebrum*	-	(2)	-	(2)	+	2
brain *cerebellum*	-	(1)	-	(1)	+	1
brain *brainstem*	-	(1)	-	(1)	+	1
*trigeminal and/or facial nerve*	-	(1)	-	(1)	-	(1)
*spinal cord and/or dura cervical*	-	(1)	-	(1)	+	1
*spinal cord and/or dura thoracic*	-	(1)	-	(1)	+	1
*peripheral nerves cervical*	-	(1)	+	1	+	1
*peripheral nerves thoracic*	-	(1)	+	1	+	1
*peripheral nerves lumbar*	-	(0.5)	+	0.5	+	0.5
*peripheral nerves sacral*	-	(0.5)	+	0.5	+	0.5
**B.2. Cardiorespiratory system**		**0.0**		**0.0**		**8.0**
*hypopharynx and/or larynx*	-	(1)	-	(1)	-	(1)
*thyroid gland*	-	(5)	-	(5)	-	(5)
*trachea*	-	(2)	-	(2)	-	(2)
*lung* right	-	(2.5)	-	(2.5)	+	2.5
*lung* left	-	(2.5)	-	(2.5)	+	2.5
heart *pericardium*	-	(1)	-	(1)	-	(1)
heart *intraventricular septum*	-	(1)	-	(1)	-	(1)
heart *four chambers*	-	(1)	-	(1)	-	(1)
heart *myocardium*	-	(1)	-	(1)	+	1
heart *valves*	-	(1)	-	(1)	-	(1)
*diaphragm* right	-	(1)	-	(1)	+	1
*diaphragm* left	-	(1)	-	(1)	+	1
**B.3. Gastrointestinal system**		**0.0**		**0.0**		**10.0**
*tongue*	-	(5)	-	(5)	+	5
*esophagus*	-	(5)	-	(5)	-	(5)
*stomach*	-	(2.5)	-	(2.5)	-	(2.5)
*intestine*	-	(5)	-	(5)	-	(5)
*rectum and/or anus*	-	(2.5)	-	(2.5)	-	(2.5)
*liver*	-	(5)	-	(5)	+	5
*gallbladder*	-	(5)	-	(5)	-	(5)
*spleen*	-	(5)	-	(5)	-	(5)
*pancreas*	-	(5)	-	(5)	-	(5)
**B.4. Genitourinary system**		**0.0**		**0.0**		**0.0**
*kidney* right	-	(2.5)	-	(2.5)	-	(2.5)
*kidney* left	-	(2.5)	-	(2.5)	-	(2.5)
*urinary bladder*	-	(5)	-	(5)	-	(5)
*prostate*	-	(5)	-	(5)	-	(5)
**or**						
*uterus*	-	(5)	-	(5)	-	(5)
external genitals *penis*	-	(2.5)	-	(2.5)	-	(2.5)
external genitals *scrotum*	-	(2.5)	-	(2.5)	-	(2.5)
**or**						
external genitals *labia*	-	(5)	-	(5)	-	(5)
**B.5. Vasculature-arteries**		**0.0**		**0.0**		**0.0**
*intracranial carotid arteries*	-	(1)	-	(1)	-	(1)
*cervical carotid arteries*	-	(1)	-	(1)	-	(1)
*mediastinal/thoracic arteries*	-	(1)	-	(1)	-	(1)
*coronary arteries*	-	(1)	-	(1)	-	(1)
*abdominal aorta*	-	(1)	-	(1)	-	(1)
*pelvic arteries*	-	(1)	-	(1)	-	(1)
*thigh arteries* right	-	(1)	-	(1)	-	(1)
*lower leg arteries* right	-	(1)	-	(1)	-	(1)
*thigh arteries* left	-	(1)	-	(1)	-	(1)
*lower leg arteries* left	-	(1)	-	(1)	-	(1)
**Total score**	** **	**9.0**	** **	**58.0**		**114.0**

The checkpoints are evaluated with “+” (structure is present) or “–”(structure is absent). Values of detected checkpoints were added at the levels of the subcategories, the two main categories and the total score. Values of checkpoints that are not present are given in brackets.

With regard to main category A (soft tissues of the head and musculoskeletal system), the yielded scores of 9.0 for the male (Aeg 777) and 55.0 for the female (Aeg 778) are rather low compared to a possible maximum score of 100.0 per individual. In contrast, the value of 87.0 for the young female (CG 33281) shows well-preserved soft tissue for category A. The largely intact musculoskeletal system including tendons, musculature, peri- and intra-articular soft tissues and intervertebral discs of the young female (CG 33281) indicates that these tissues did not undergo decay and disconnection, hence suggesting a careful mummification procedure. Very low scores of 0.0 for the male (Aeg 777) and 3.0 for the female (Aeg 778) are yielded for main category B (organs and organ systems), considering a possible maximum score of 100.0 per specimen. The higher value of 27.0 for the young female (CG 33281) shows that organs and organ systems are better preserved. Several organs could be identified, including the brain, remnants of the cardiorespiratory system (lungs, myocardium, diaphragm), and remnants of the gastrointestinal system (tongue, liver). Tissues of the genitourinary system and the vasculature-arteries were not preserved in any of the mummies.

### Foreign objects

**Fig 16:**
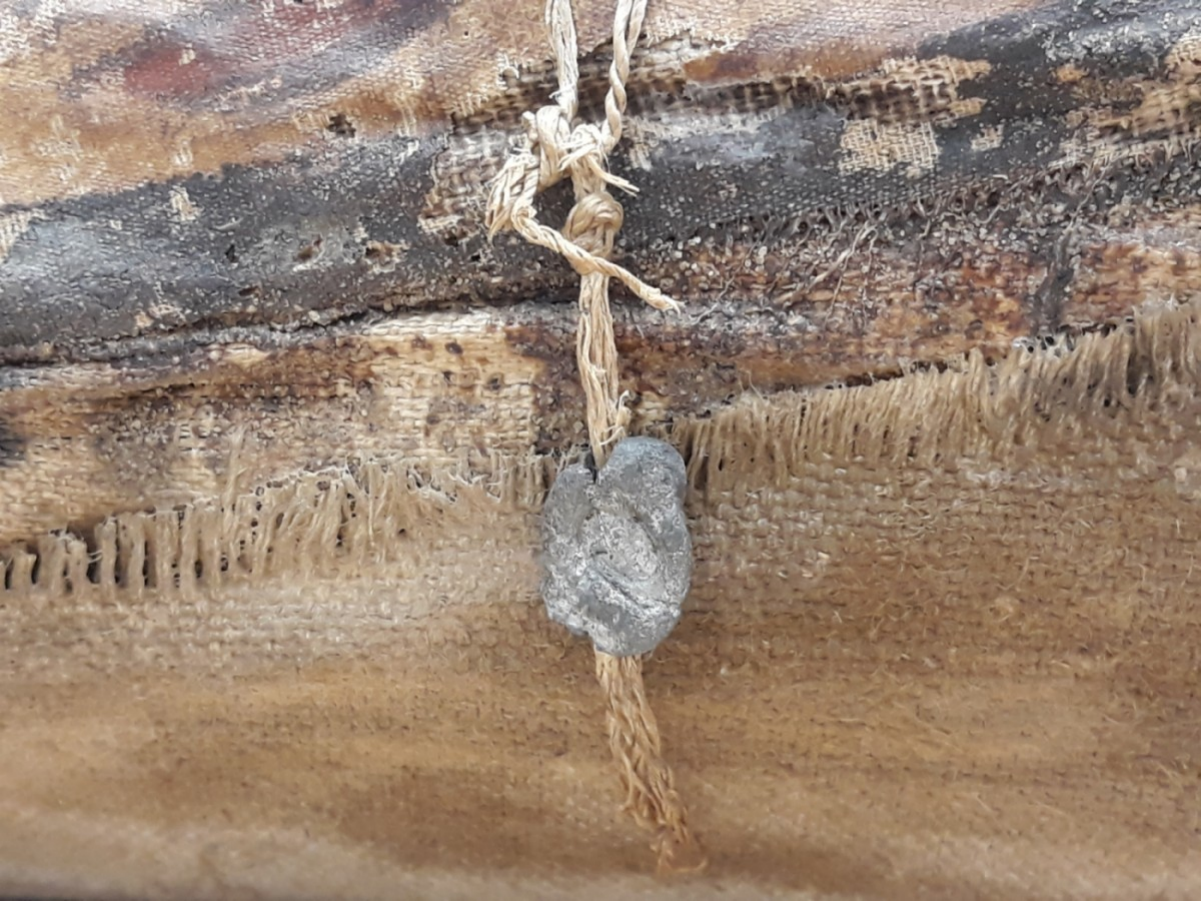
Male mummy (Aeg 777). The detail photo shows a lead seal at the outermost textile layer on the left side of the mummy. The seal is fixed at the level of the mummy’s thigh with a cord about 15 cm in length. An almond-shaped depression in the center of the seal is most probably the insignia of the mummification workshop (© Sculpture Collection, Dresden State Art Collections, photos: M. Gander/M. Loth).

The male’s body (Aeg 777) is placed on a wooden board (width 18 cm, depth 2.3 cm), broken in several areas (Fig 10B). Two radiopaque metal objects (HU ≥ 3071) of similar size and shape were detected inside the mummy. One metal object is visible dorsally inside the left thorax (length (superior/inferior) 1.8 cm, width (right/left) 1.5 cm, depth (anterior/posterior) 1.6 cm) (Fig 11). The second metal object was observed medial to the right femur (sup/inf 2.0 cm, rt/lt 1.3 cm, ant/post 1.3 cm) (Fig 10A). A metal (lead) seal (sup/inf 1.6 cm, rt/lt 2.2 cm, ant/post 1.9 cm) is fixed on the outermost textile layer on the left side of the mummy (Fig 16). The two metal objects of unknown identification inside the body are suggested to be metal seals as well, since they correspond in size and density to the known metal seal on the outermost textile layer.

**Fig 17:**
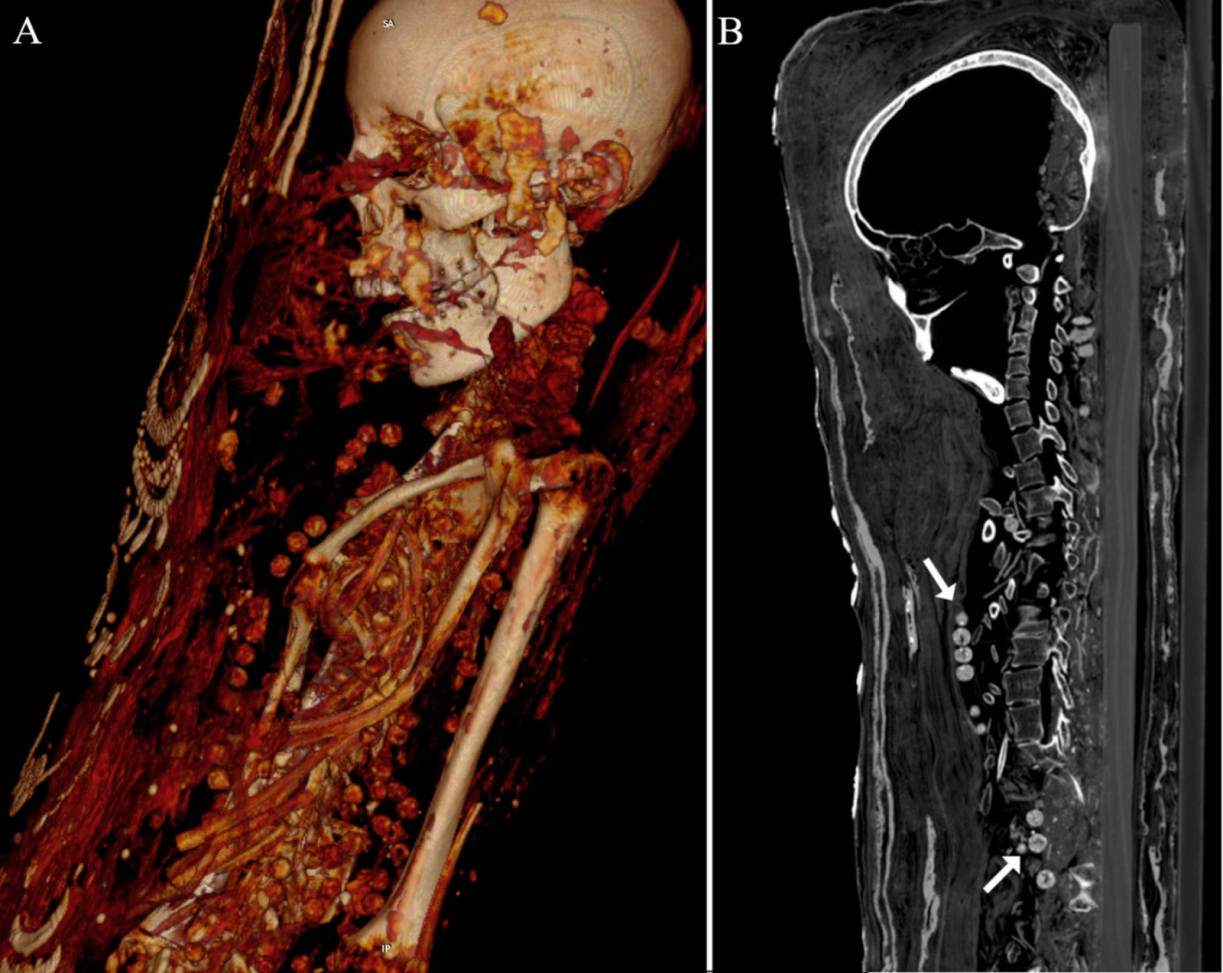
Female mummy (Aeg 778). (A) volume rendered reconstruction illustrates numerous scattered beads in the thoracic region; (B) sagittal maximum intensity reconstruction shows a few of the perforated beads in detail (arrows).

The female’s body (Aeg 778) is placed on an intact wooden board (length 158 cm, width 15.4 cm, depth 1.9 cm) (Fig 12B). Numerous perforated circular beads (diameter 1 cm) were identified scattered in the thoracic region (Fig 17). Their rather low radiodensity (HU ≈ 180 ± 15) likely represents organic material, such as animal horn of HU ≈ 268 ± 15.1 [61].

Two elongated metal foreign objects (HU ≥ 3071, length 3.6 cm), each showing a small rounded structure (diameter 0.5 cm) on one end, were found inside the left abdominal region (Fig 12A). Their shape and radiodensity most probably indicate metal nails. Two circular metal foreign objects (HU ≥ 3071) of similar size and shape were detected inside the mummy’s wrappings. One of the circular objects is placed close to the right hand (sup/inf 2.8 cm, rt/lt 1.8 cm, ant/post 2.8 cm) and the second one is visible between the femurs (sup/inf 3.1 cm, rt/lt 3.0 cm, ant/post 2.4 cm). The authors suggest these objects represent coins or medallions.

An intact wooden board (exact length undetermined, width 16.7 cm, depth 1.9 cm) was detected beneath the young female`s body (CG 33281) (Fig 13B). The upper segment of the stuccoed shroud beyond the mummy’s head was not scanned making the exact length of the enclosed mummy board unknown. However, the mummy board is seen to extend the entire length of the mummy from the head to the talocalcaneal joint of the ankle, with an overall estimated length of the mummy board to be between 150 to 153 cm. Seven irregular shaped radiopaque metal seals (HU ≥ 3071) of similar size (2 cm) are attached with cords on the outermost textile layer at several body regions, including head (one seal), thorax (one seal), pelvis (two seals), lower legs (one seal), and feet (two seals).

**Fig 18:**
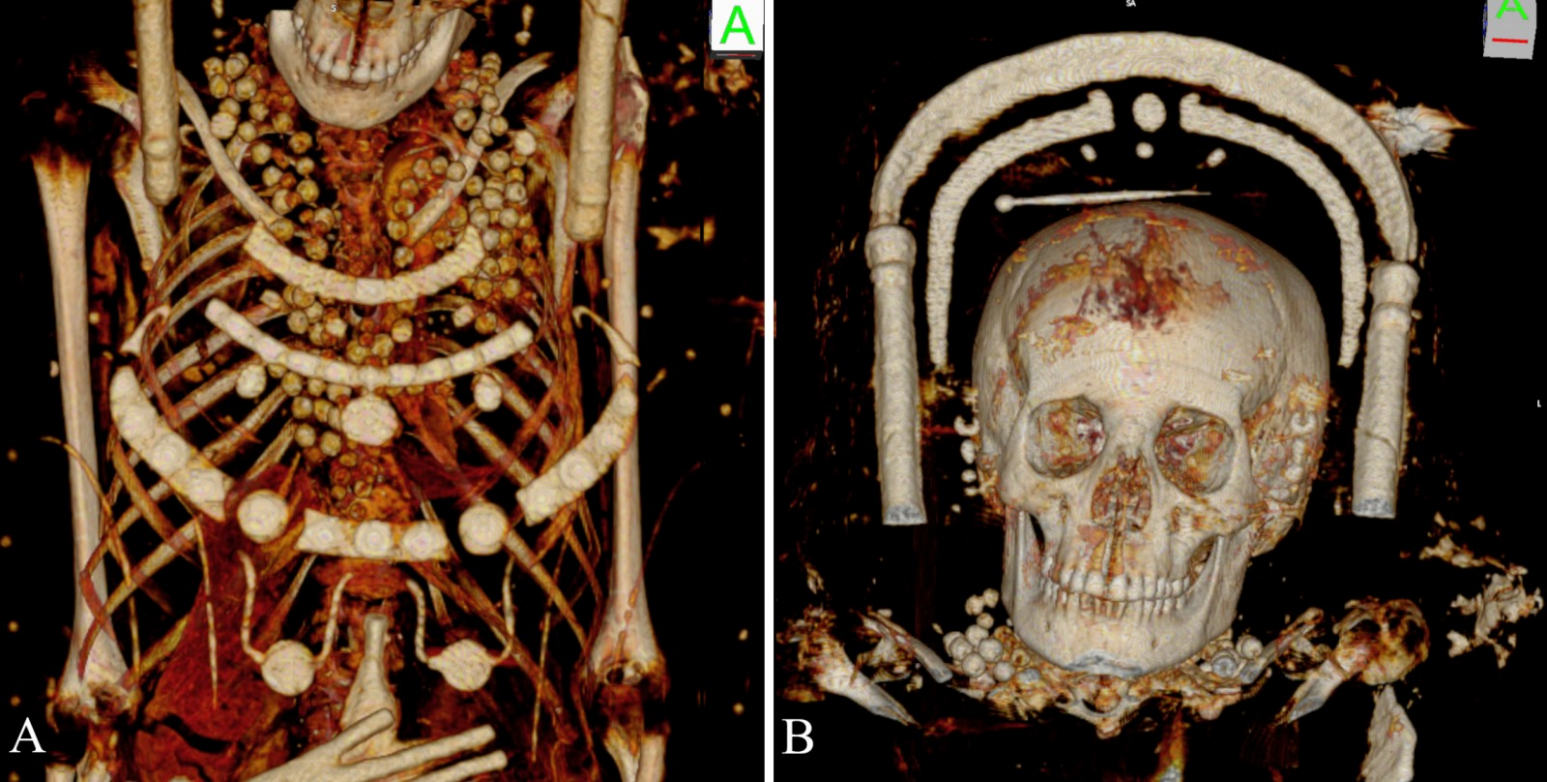
Volume rendered reconstructions representing details of the young female mummy (CG 33281). (A) numerous beads around the neck and on the thoracic region, suggesting an intact necklace/or several necklaces; (B) a hairpin on top of the head indicating an upswept hairstyle.

Different types of perforated beads were identified inside the mummy’s wrappings. These include longitudinal thin-walled low density beads of very small size (length 1.5 cm, width 0.4 cm) between the innermost textile layers at the thorax (Fig 14) and numerous perforated circular beads (diameter 1 cm, HU ≈ 665 ± 358) around the neck and on the thorax (Fig 18A). The circular beads are likely still attached to each other, suggesting an intact necklace/or several necklaces. A hairpin (length 9.1 cm) with a small rounded structure (diameter 0.6 cm) at one end is visible on top of the head (Fig 18B). It is plausible to assume that the young female’s hair was upswept at the time of burial, resembling the hairstyle depicted on the mummy portrait. The radiodensity of the hairpin (HU ≈ 1692 ± 129) indicates bone, antler or semi-precious stones, considering the comparative values cited in [61].

### Age at death, sex and body height

The evaluation of the morphological features of the skull and pelvis of the mummy with male portrait (Aeg 777) confirmed a male inside the wrappings. Dental status and skeletal markers indicate an adult between 25 to 30 years. The third molars have fully erupted. All teeth show a less advanced dental wear. The suggested age at death is further supported by the presence of unfused cranial sutures, fused epiphyses and a high trabecular bone density of long bones. Body height was estimated about 162.5 ± 3.4 cm [59] and about 164.7 ± 3.1 cm [60].

Concerning the mummy with female portrait (Aeg 778) in Dresden, the estimation of sex-specific markers of the skull and the pelvis confirmed a female individual. Dental status and skeletal markers indicate an age at death between 30 to 40 years. Dentition is complete and shows a regular moderate dental wear. The fusion of cranial sutures is more advanced compared with the male, and the trabecular bone density of the long bones is moderate. The estimated body height is about 148 ± 3.5 cm [59] and about 151.6 ± 3.8 cm [60].

The evaluation of sex-specific markers of the mummy with female portrait (CG 33281) in Cairo confirmed a female, as indicated by the portrait. The presence of a complete dentition without significant dental wear, unfused cranial sutures and partially unfused epiphyses suggested an age at death between 17 to 19 years. In particular, the unfused distal ulnar and radial epiphyses indicated a slightly younger age than 19 years that was published by Allam and colleagues [52]. Body height was reconstructed about 152.7 ± 3.5 cm [59] and about 156.6 ± 3.8 cm [60].

### Anatomical anomalies and paleopathological findings

**Fig 19:**
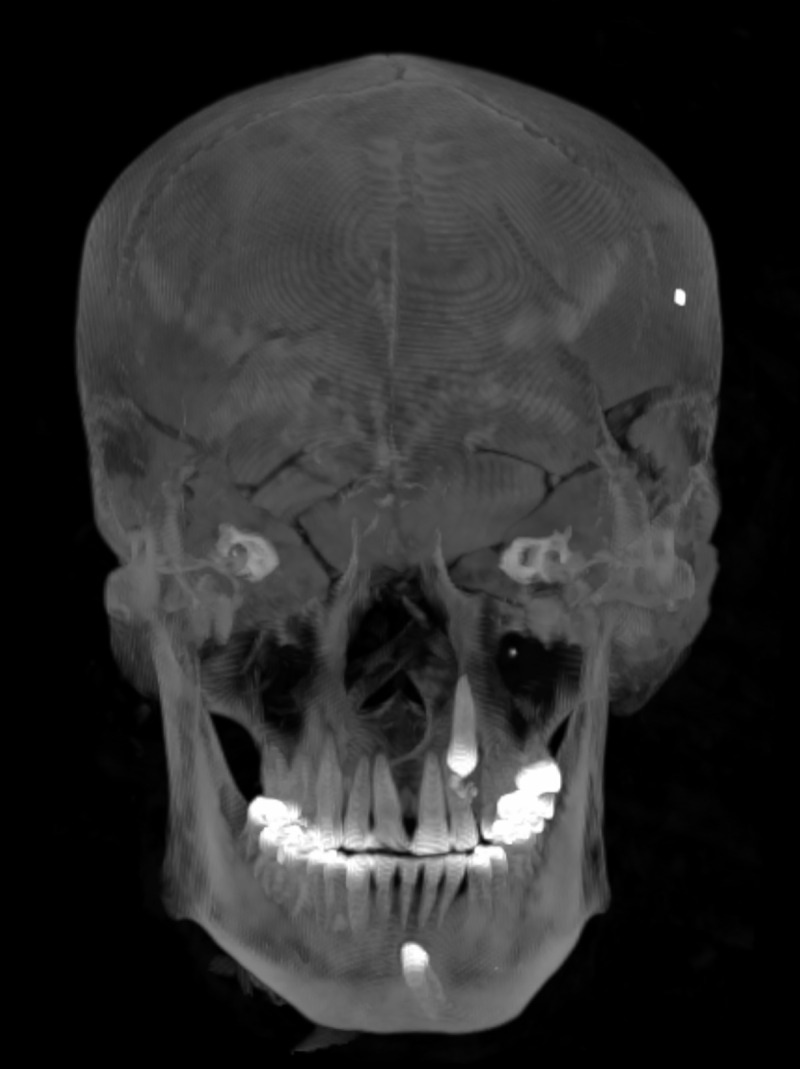
Skull of the male mummy (Aeg 777). 3D maximum intensity projection visualizes the unerupted permanent maxillary and mandibular left canines as a congenital dental anomaly.

The investigation of the male (Aeg 777) revealed congenital dental anomalies; two unerupted permanent teeth, maxillary and mandibular left canines (Fig 19). Remnants of a retained deciduous tooth, most probably a canine, was detected at the position of the unerupted permanent maxillary left canine. Carious lesions were found on two teeth; the maxillary right first molar whose crown was almost completely absent and associated with a periapical abscess, and the maxillary left second molar with a less pronounced carious lesion medially. It is assumed, that the antemortem loss of the mandibular left first molar might have resulted from caries as well. Schmorl’s nodes were detected on several vertebral bodies of the lower thoracic and lumbar spine.

**Fig 20:**
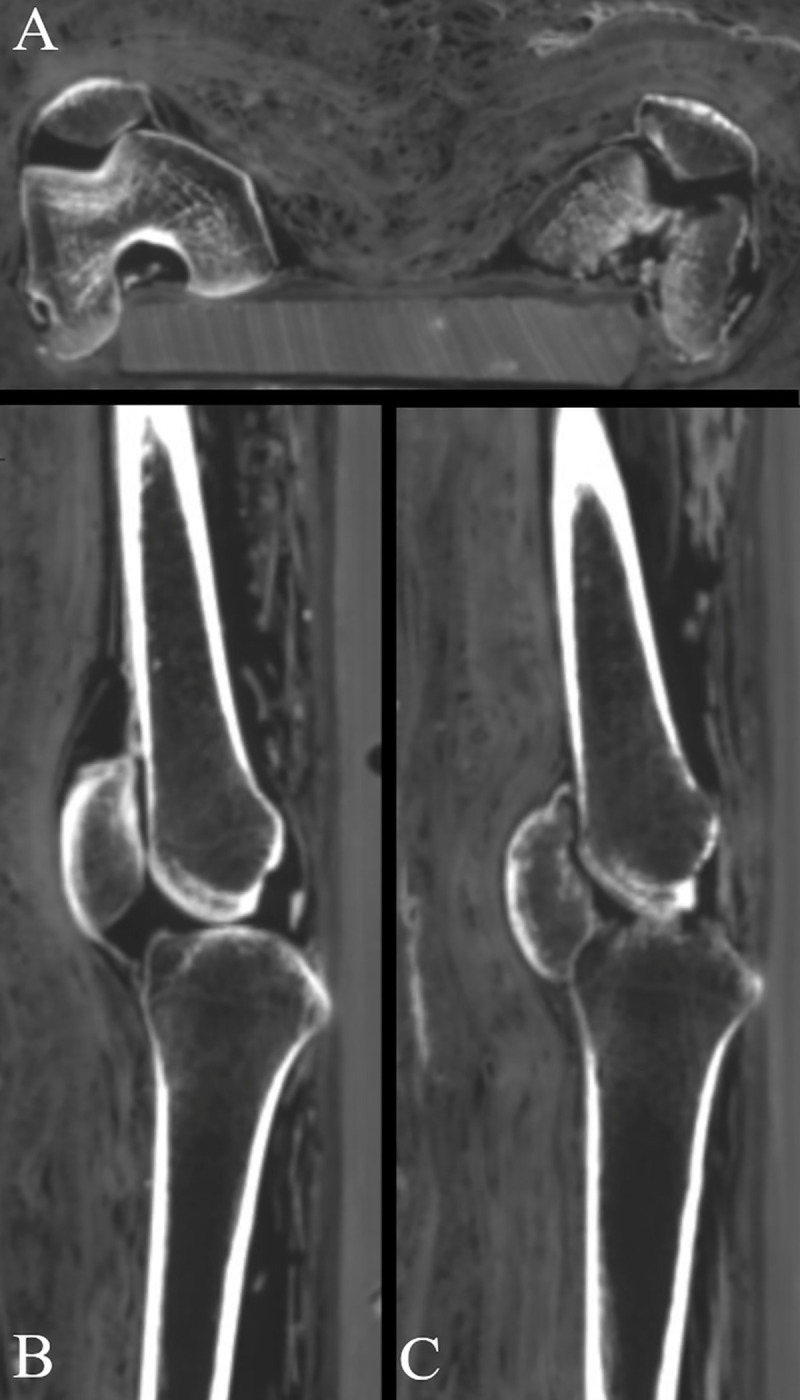
Multi-planar reconstructions visualizing the knees of the female mummy (Aeg 778). (A) both knees in axial view with evidence of juxtaarticular osteoporosis in the left knee; (B) the right knee joint in sagittal view without any pathological changes; (C) the left knee joint in sagittal view with evidence of arthritis, such as lucencies of the subchondral bone in the femorotibial and femoropatellar joints, and the patella subluxed inferiorly, contacting the proximal tibia.

The spine of the female (Aeg 778) shows a pronounced scoliosis, convex left in the thoracic segment and convex right in the lumbar segment, either existing during lifetime or occurring during post mortem manipulations. Several Schmorl’s nodes and irregularities on the endplates of the vertebral bodies were detected at the thoracic and lumbar segments of the spine. The left half of the pelvis, whose skeletal elements are partially broken, is placed slightly more cranially than the right half of the pelvis. Evidence of advanced arthritic changes were identified at the left knee joint (Fig 20). This includes lucencies of the subchondral bone of the femorotibial joint and of the femoropatellar joint as well as juxtaarticular osteoporosis. The patella is subluxed inferiorly and the quadriceps tendon is largely interrupted.

**Fig 21:**
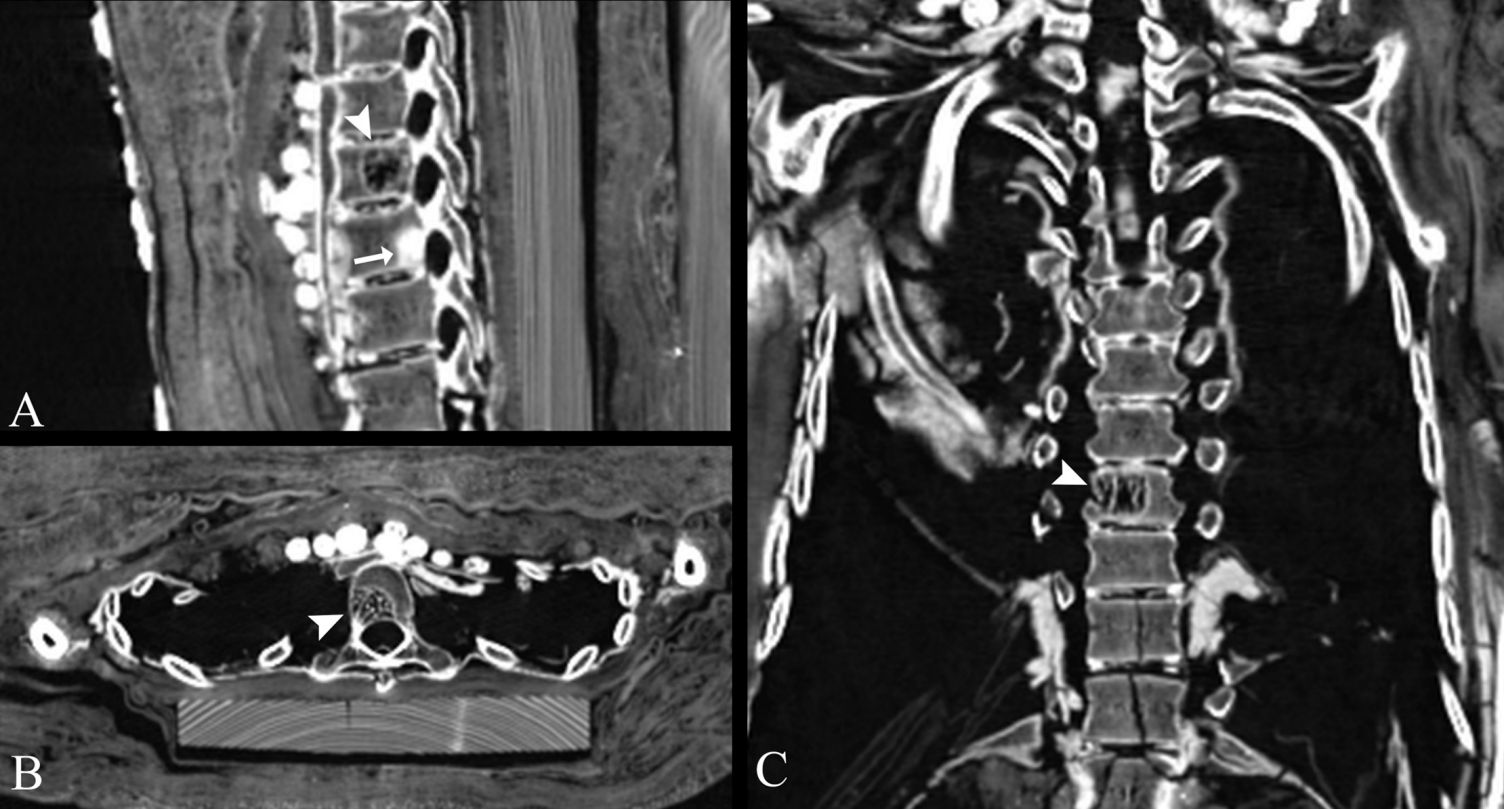
Segment of the thoracic spine of the young female mummy (CG 33281). Multi-planar reconstructions show a focal area of coarsening of the vertical trabeculae without expansion of the cortex, indicating a vertebral hemangioma of T8 thoracic vertebra in (A) sagittal, (B) axial, and (C) coronal views (arrow heads). Note a small-size osteoma (arrow) dorsally within the trabecular bone of T9 thoracic vertebra in (A).

The young female (CG 33281) shows a focal area of coarsening of the vertical trabeculae without expansion of the cortex on the right side within the T8 thoracic vertebra (diameter 1.5 cm), which most likely represents a vertebral hemangioma (Fig 21). This so-called *honeycomb* or *corduroy* pattern is considered pathognomonic for vertebral hemangiomas [62, 63]. A small-size osteoma was detected dorsally within the trabecular bone of T9 thoracic vertebra (Fig 21A).

Although CT investigation revealed several anatomical anomalies and paleopathological changes, the cause of death could not be identified in any of the specimens investigated.

## Discussion

### Decoration on the mummy shrouds

As described by Filer [21], radiological investigations sometimes yield discrepancies concerning the sex or age at death of a portrait mummy when compared with the facial features implied by the portrait. According to Wilkinson [64], it is still discussed in research whether mummy portraits reflect lifelike or idealized illustrations of the dead as well as whether the portrait was produced posthumously or when the person was alive. Researchers have investigated this aspect through the creation of facial reconstructions based on skulls or skull replicas in a blind approach of several mummies and have compared them with their portraits with varying results [64]. With regards to the current study, the results of sex determination and age at death estimation of the mummies are consistent with the sex and biological age illustrated on the mummy portraits. However, making a digital facial reconstruction based upon generated CT data might be recommended to clarify whether these are lifelike or idealized representations of the deceased.

The mummy shrouds show various decorative elements and religious motifs from both Graeco-Roman and ancient Egyptian tradition; some of which will be discussed in the following. Graeco-Roman features include the golden wreaths, hairstyles, garments, jewelry, mummy garlands and vessels depicted in the hands. The representation of mummy portraits showing the deceased as living persons, *clavi* and the linked necklace made of minted gold foil visible on the middle-aged female (Aeg 778) are associated with Roman tradition. The golden wreaths depicted on the heads might be so-called *wreaths of justification*, which are discussed by Egyptologists as possible symbols for the successful verification of the deceased through the judgment of the dead and transition into the underworld [32]. The Gorgon heads on the females’ shrouds are thought to serve a protective function similar to the head of the gorgon Medusa, which is illustrated on the shields of the Greek god Zeus and the goddess Athena. The suggested illustration of two ears of corn and a poppy seed-pod in the left hand of the middle-aged female (Aeg 778) are associated with the Greek goddess of harvest and agriculture, Demeter, who is identified with the Egyptian goddess Isis, the wife of Osiris [65], and with the Egyptian goddess of harvest, Thermouthis [32]. The Gorgon heads as well as the ears of corn and a poppy seed-pod are also depicted on a similar female shroud (ÄM 11659) housed in the Egyptian Museum and Papyrus Collection, Berlin. The illustrations of mummy garlands on the shrouds of the male (Aeg 777) and the young female (CG 33281) indicate similarities of both Greek and Egyptian tradition. Depicting wreaths and garlands with the deceased, as frequently shown on Roman Period mummy masks and portraits, is assumed to originate from Greek tradition. Large amounts of aromatic plants were discovered in Graeco-Roman cemeteries. However, decorating mummies with garlands made of blossoms, leaves and fruits is also evidenced as part of the Egyptian burial practice from the 18^th^ dynasty (c. 1550−1295 BC) [15] onward [66]. According to Egyptian afterlife concept, lovely fragrances were important, since a magical effect was attributed to them facilitating the enlivening of the deceased [24, 32].

The stylized representation of a bead-net as a symbol of the successful transition of the deceased into the underworld point to the Osirian afterlife concept in which every deceased becomes similar to the god Osiris [36, 67], who was depicted dressed in a bead-net at least since the late New Kingdom (c. 1550−1069 BC) [15]. From the Late Period (664−332 BC) [15] onward, mummies were frequently covered by an actual bead-net consisting of blue-green tubular beads commonly made of faience with polychrome motifs and symbols, such as the four Sons of Horus, protecting the internal organs, and the winged scarab, representing rebirth and regeneration. In the Roman Period, representations of bead-nets were often painted directly on the shroud [67]. Representations of the Apis bull that are shown on both female mummies, are also seen on a similar female shroud (CG 33280) housed in the Museum of Egyptian Antiquities, Cairo, and on a female shroud (ÄM 11659) in the Egyptian Museum and Papyrus Collection, Berlin. The Apis bull points to the Apis cult, which was the most important cult at the city of Memphis and the Saqqara necropolis. The Apis bull was worshipped as the embodiment of the Egyptian god Ptah, a powerful creator-god and patron of the craftsmen. After his death, the Apis bull was mummified and buried with great expense in the so-called *Serapeum* at Saqqara [2, 68, 69]. Hence, the representation of the Apis bull on the shrouds of the females clearly refers to the local cult and suggests a particular relationship of the deceased to the Memphite region.

### Artificial mummification and preservation

As the skeletal integrity of the male (Aeg 777) and the female (Aeg 778) are partially damaged and their soft tissue preservation is poor, suggestions on the technique of body treatment applied for artificial mummification should be considered carefully. However, the findings indicate that an extensive procedure of mummification characterized by the removal of brain and internal organs as well as the use of large amounts of embalming liquids is unlikely to have been carried out. This is also supported when taking into account the much better-preserved body of the young female (CG 33281) whose brain and several internal organs were identified. Therefore, the authors assume that the specimens might have been mummified largely as a result of soft tissue dehydration by applying large amounts of natron, commonly used for the desiccation of bodies in ancient Egypt, without excerebration or evisceration.

It is suggested in several publications that during the Graeco-Roman Period the beautiful external appearance of a mummy was more important than the preservation of the body itself, resulting in a decline of the technique of artificial mummification [2, 3]. However, several authors disagree with the idea of a general decline of mummification craftsmanship during the Graeco-Roman Period [17, 19, 28]. They point to various factors that may have played a role, such as costs, different methods and quality standards, and varying local burial customs [17, 19]. Detailed investigations and suggestions on the tradition of excerebration and evisceration in Egypt are described in Wade and colleagues [70] and Wade and Nelson [71]. A study by Loynes [28] revealed the presence of brain in four cases and the presence of intestines in eight cases, during an investigation of 17 Roman Period specimens. Loynes interpreted the increasing lack of excerebration and evisceration during the Roman Period as evidence for changing, however not declining practices of artificial mummification at that time [28]. According to Bowman [72], demographic growth up to an estimated population size of about seven million is suggested for the Roman Period. As a consequence of population growth, increasing demands on artificial mummification are assumed to have arisen, which could have led to urgent and incomplete procedures of body treatment [72]. It should further be considered that soft tissue might also have been in an advanced state of decomposition before the body was wrapped, as suggested for a Graeco-Roman child mummy in wrappings investigated by Rosendahl and colleagues [73].

Bone fractures and skeletal dislocations which were observed on all specimens, although to a greater extant on the mummies housed in Dresden, were most likely caused during excavation and transport. Concerning the male (Aeg 777), several indications suggest that the mummy was opened soon after discovery. These include a paragraph in Della Valle’s travelogue mentioning he saw the corpse of a naked mummy [40] as well as the identified seals inside the body and the damage of wrapping on the backside. The flattened shape of the upper body of the young female (CG 33281) might result from dehydration and/or tight textile wrapping.

### Foreign objects

The presence of a wooden board beneath the body, evidenced for all specimens investigated in this study, is known from numerous Roman Period mummies, especially those wrapped with mummy shrouds [20, 22, 23, 27, 28].

The suggested identification of two metal seals inside the body of the male (Aeg 777) is supported by Della Valle’s travelogue, as it mentions several lead seals attached to the mummy [40]. In a detailed analysis of the mummy shroud and textile wrapping by Dötzel in 2004 (S1 Appendix), the author describes six punctures in the shroud (two of them with remnants of cords) and an area where textile is absent. As one seal is still visible, the observations indicate that originally eight seals must have been present on the shroud. It is therefore plausible, that two of them were translocated inside the body during the mummy opening at the time of discovery; the whereabouts of the other seals are unknown.

The identification of bead jewelry, most likely made from organic material, on both females is attractive, however not unique, since such items were occasionally found on other unrelated mummies [74, 75]. Materials known to be used for the production of beads in Graeco-Roman Egypt are glass, hard stone, glazed stone, soft stone, metal, and miscellaneous materials like amber, black resin, ostrich shell and wood [76].

The presence of metal nails inside the abdomen of the middle-aged female (Aeg 778) is without a plausible explanation. Nails are broadly found in the archaeological record, for example at the cemetery of Tuna El-Gebel. However, such findings are usually made together with other metal objects that might have been applied to furniture used as grave goods [77]. The identification of two other metal objects suggested to be coins or medallions next to the female’s right hand and femur (Aeg 778) are interesting radiological findings. Providing the deceased with coins is today broadly identified as so-called *Charon‘s obol*, originating from Greek tradition. However, further suggestions regarding the use of coins in ancient funerary practice within a broader context are given in Stevens [78]. With regards to Egypt, the tradition of burying the deceased provided with money was not evidenced before the 3^rd^ quarter of the 4^th^ century BC. Coins have been found near the head, within the mouth and hands, between the bandages as well as beneath, near or on top of the body [79]. However, these items might also be medallions, probably similar to the Gorgon's head depicted as a pendant on the females’ shrouds. Some necklaces with Gorgon's head medallions are preserved from Egypt, such as one item from the late 2^nd^ to the 3^rd^ century AD (GRA 1917.6–1.2737, housed in the British Museum [34]).

The detection of a hairpin with the young female (CG 33281) through CT investigation is remarkable, as hairpins are assumed to be depicted on the portrait as well. They are frequently visible on female mummy portraits. Other sources providing information on the use of hairpins include female representations on tomb walls and archaeological findings, mostly found with female burials. Hairpins are commonly made of bone, ivory, wood, steatite, glass, gold, silver, and bronze [80]. Two hairpins were detected, for example, attached to the Roman Period mummy of a young female excavated at Antinoopolis (no. 90001594, housed in Musée d’histoire naturelle Guimet) [27].

### Age at death, sex and body height

Generated demographic profiles of ancient Egyptian populations are rather young, showing high infant mortality and low life expectancy at birth [81, 82]. The most comprehensive study on human remains from the Roman Period was carried out in the Dakhleh Oasis Project. According to Dupras and colleagues [83], 727 individuals from the Kellis 2 cemetery (c. 100−450 AD) included 64% juveniles (< 15 years) and 36% adults. Most specimens (24.5%) died between birth and one year and the second largest group (17.6%) died between 16 to 35 years. Body height reconstruction of individuals from Kellis 2 revealed about 166 cm for males and approximately 156 cm for females. The results on age at death and body height suggested for the specimens investigated in the current study are similar to the results on individuals from the Kellis 2 cemetery.

### Health status

Carious lesions and a periapical abscess were revealed during the CT investigation of the male mummy (Aeg 777). One might suppose a lower consumption of carbohydrates in the diet and/or better oral hygiene for the females investigated, achieving an outstanding dental health status. The informative value is limited however, due to the small number of individuals analyzed. A study of Miller on Egyptian human remains [84] concluded that from the Late Period onward specimens more frequently showed occlusal and interstitial caries than root carious, probably caused by an increased proportion of sucrose in the diet, especially sweeteners such as honey, dates, figs and fruit juices. High carious rates were also determined in a study on individuals dating from the New Kingdom until the Late Period (1550−332 BC) [15] from the “Tombs of the Nobles” in Thebes-West [85]. As described by Lanfranco and Eggers [86], several events can be mentioned from the past concerning the advent of starchy types of food and sugar in the European diet, combined with increased caries incidence identified through bioarchaeological investigations of human remains. These events include the increasing production of flour and intake of sugar cane since the 16^th^ century, the import of corn, potatoes, cocoa, sugar etc. from America to Europe from the 17^th^ century onward, the increasing production of refined sugar and the advent of flour mills in the 18^th^ century, as well as the growing consumption of sugar and refined carbohydrates during the second half of the 19^th^ century [86].

The clinical presentation of Schmorl’s nodes on the endplates of vertebral bodies as seen in the male (Aeg 777) and middle-aged female (Aeg 778) are typically asymptomatic incidental findings. Kyere and colleagues [87] described a number of theories that have been proposed by researchers on the pathogenesis of Schmorl’s nodes; however, no consensus currently exists.

Evidence of advanced arthritis in the left knee joint of the female (Aeg 778) is suggestive of post-traumatic arthritis or possibly septic arthritis, such as described in Shirtliff and Mader [88]. The slightly misaligned left half of the pelvis might be a consequence of the thoracolumbar scoliosis and/or attempt to reduce mechanical pressure on the painful left knee joint.

Vertebral hemangioma suggested for the young female (CG 33281) is a common benign tumor in the spine more frequently observed in individuals beyond the fourth decade of life [89]. Most hemangiomas of the vertebral column are asymptomatic and discovered incidentally. Symptoms occur when the lesion in an affected vertebra compresses the nerve roots or spinal cord secondary to epidural extension. Occasionally, a pathologic fracture can occur [62, 63]. As the hemangioma did not develop beyond the margins of the vertebral body, and as no fracture was present, clinical symptoms would have been unlikely in the young female. Vertebral hemangiomas are rarely described in studies on mummies [11, 90] and skeletons from archaeological contexts [91]. Molto and colleagues [91] published the case of a Roman Period female between 50 to 65 years from Kellis 2 cemetery. The skeleton showed expanded and lysed cortex and thickened vertical trabeculae of T12 thoracic vertebra which the authors interpreted as the *corduroy* pattern of vertebral hemangioma.

## Conclusions

Combining the iconographic analyses of the decoration of the attached mummy shrouds with the paleoradiological investigation of the mummified bodies inside offered insight on contemporary afterlife concepts and the tradition of artificial mummification, as well as insight on the bioanthropological profiles of the specimens. The elaborately decorated shrouds with mummy portraits, gilded stucco elements and religious motifs of Graeco-Roman and ancient Egyptian conventions indicated an upper socioeconomic status of the deceased. The usage of CT technique enabled a non-destructive visualization of the internal mummy structures, including the bodies themselves and accompanying foreign objects, in high-resolution images. Particularly noteworthy are the detection of wooden boards, non-metallic bead jewelry and a hairpin, as well as the suggested identification of lead seals, nails and two coins or medallions. The individuals died at rather young ages and suffered several physical stresses, such as caries and arthritis, however, their cause of death could not be determined. The three stucco-shrouded portrait mummies represent unique examples of the artificial mummification tradition practiced at the Saqqara necropolis at the end of the Roman Period. They are moreover considered as valued cultural heritage due to their exceptional collection history and intact preservation.

## Supporting information

S1 Appendix List of used unpublished reports. (DOCX)
