# Supplementary material for: Decorated bodies for eternal life: A multidisciplinary study of late Roman Period stucco-shrouded portrait mummies from Saqqara (Egypt)
Source: PLoS One. 2020 Nov 4;15(11):e0240900. doi: 10.1371/journal.pone.0240900 (PMC7641350; doi:10.1371/journal.pone.0240900)
Supplement: S1 Appendix — (DOCX) [file pone.0240900.s001.docx]

**S1 Appendix. List of used unpublished reports**

Begoff P. Röntgenologische Beurteilung der beiden della Valle-Mumien. Radiologische Klinik des Bezirkskrankenhauses Dresden-Friedrichstadt. 1988.

Dötzel F. Technologische Untersuchung an zwei ägyptischen Portraitmumien aus dem Bestand der Staatlichen Kunstsammlungen Dresden, Skulpturensammlung, Inventarnummer Aeg. 777 und 778, Saqqara, 1. Hälfte des 4. Jahrhundert n. Chr. Hochschule für Bildende Künste, Dresden. Seminar work, 2004.

Germer R. Materialsammlung zu den Dresdner Mumien Aeg 777 und Aeg 778. 2015.

Pahl WM. Gutachten über die ägyptischen Mumien Inv. Aeg 777 und 778 der Staatlichen Kunstsammlungen Dresden (DDR). 1990.

By the courtesy of the Dresden State Art Collections
